# A genetic sum score of effect alleles associated with serum lipid concentrations interacts with educational attainment

**DOI:** 10.1038/s41598-021-95970-z

**Published:** 2021-08-16

**Authors:** Carina Emmel, Mirjam Frank, Nico Dragano, Markus M. Nöthen, Raimund Erbel, Karl-Heinz Jöckel, Börge Schmidt

**Affiliations:** 1 Institute for Medical Informatics, Biometry and Epidemiology, University Hospital of Essen, University of Duisburg-Essen, Hufelandstraße 55, 45147 Essen, Germany; 2 Institute of Medical Sociology, Centre for Health and Society, University Hospital Düsseldorf, Düsseldorf, Germany; 3 Institute of Human Genetics, University of Bonn, Bonn, Germany; 4 Department of Genomics, Life and Brain Center, University of Bonn, Bonn, Germany

**Keywords:** Risk factors, Epidemiology

## Abstract

High-density lipoprotein cholesterol (HDL-C), low-density lipoprotein cholesterol (LDL-C), and total cholesterol (TC) levels are influenced by both genes and the environment. The aim was to investigate whether education and income as indicators of socioeconomic position (SEP) interact with lipid-increasing genetic effect allele scores (GES) in a population-based cohort. Using baseline data of 4516 study participants, age- and sex-adjusted linear regression models were fitted to investigate associations between GES and lipids stratified by SEP as well as including GES×SEP interaction terms. In the highest education group compared to the lowest stronger effects per GES standard deviation were observed for HDL-C (2.96 mg/dl [95%-CI: 2.19, 3.83] vs. 2.45 mg/dl [95%-CI: 1.12, 3.72]), LDL-C (6.57 mg/dl [95%-CI: 4.73, 8.37] vs. 2.66 mg/dl [95%-CI: −0.50, 5.76]) and TC (8.06 mg/dl [95%-CI: 6.14, 9.98] vs. 4.37 mg/dl [95%-CI: 0.94, 7.80]). Using the highest education group as reference, interaction terms showed indication of GES by low education interaction for LDL-C (ß_GES×Education_: −3.87; 95%-CI: −7.47, −0.32), which was slightly attenuated after controlling for GES_LDL-C_×Diabetes interaction (ß_GES×Education_: −3.42; 95%-CI: −6.98, 0.18). The present study showed stronger genetic effects on LDL-C in higher SEP groups and gave indication for a GES_LDL-C_×Education interaction, demonstrating the relevance of SEP for the expression of genetic health risks.

## Introduction

Elevated serum concentration of low-density lipoprotein cholesterol (LDL-C) is an important causal risk factor for cardiovascular disease (CVD)^1–3^ and has now largely replaced total cholesterol (TC) as the primary treatment target for dyslipidemia^1^. Reduced serum concentration of high-density lipoprotein cholesterol (HDL-C) is independently associated with CVD^4,5^, in genetic studies however, HDL-C has not been causally associated with CVD^3^. Serum lipid concentrations (in the following named as lipids) are complex human traits that are influenced by both genetic and lifestyle factors^6^. With regard to health inequalities, research has shown that indicators of socioeconomic position (SEP) such as educational attainment, household income and employment status are also associated with lipids^7–11^. However, the association has been of different strength with regard to different SEP indicators, heterogeneous between men and women and appears to be of different direction in developed and developing countries. In developed countries a more unfavorable serum lipid profile (higher LDL-C and TC levels and lower HDL-C levels) is observed in lower SEP groups^8,10–13^.

The genome-wide association study (GWAS) meta-analysis from the Global Lipids Genetics Consortium (GLGC) in 2013 identified 157 loci associated with lipids accounting for up to 12% of the variance in each lipid trait^14^. Subsequent large-scale GWAS demonstrated enhanced gene discovery from expanded sample sizes, independently reporting additional novel loci in different ancestry groups, accounting for up to 20% of the variance in lipids^15–17^. In contrast family-based association studies have indicated that 30–70% of the variance in lipids is genetically based^18,19^.

It is assumed that gene-environment (G × E) interactions, where the effect of some gene variants depend on specific environmental exposures^20^, may account for parts of the unexplained variance of lipids^21^. G×E interactions for lipids have been observed for life style risk factors such as obesity, smoking, diet, physical activity, diabetes and sleep duration^22–29^. While some studies showed stronger effects of lipid-increasing alleles in groups reporting a more favorable life style such as lean or physically active individuals^27,28^, Bentley et al. also reported SNPs with stronger effects in smokers as an example for an unfavorable life style^24^. So far interactions of lipid-related effect alleles with SEP have not been investigated in adults, but G×SEP interactions on overall health and other traits such as body mass index (BMI) have been indicated by twin and population based studies^30–32^. Li et al. reported an interaction between one SNP related with a metabolically obese, normal weight phenotype including high TC levels and a composite score including parental education and household income on metabolically obese, normal weight children^33^.

SEP can be considered as a context-defining variable that describes certain risk constellations such as unequally distributed environmental, psychosocial and behavioral health risk factors and may be better suited for describing health-related environments as a whole rather than single risk factors. Thus, investigating interactions between lipid-associated loci and SEP indicators may be crucial for identifying subgroups for which genetic effects show stronger signals than for the average population^34^ and who may benefit from genotype-based targeted intervention^35^.

The aim of this study was to investigate whether the SEP indicators education and income interact with genetic sum scores of lipid-increasing effect alleles (GES) for HDL-C (GES_HDL-C_), LDL-C (GES_LDL-C_) and TC (GES_TC_) in a population-based cohort study. In order to explore whether any detected SEP interactions can be explained by SEP-associated life style risk factors, information on smoking, BMI, physical activity, alcohol consumption and diabetes mellitus was included in the interaction analysis. Main results of GES based on the GLGC GWAS meta-analysis^14^ were compared to the results of GES also including loci of more recently published GWAS to check for differences in lipid prediction and GES×SEP interaction.

## Methods

### Study population

All analysis are based on baseline data of the Heinz Nixdorf Recall Study (Risk factors evaluation of coronary calcium and lifestyle cohort), a population-based prospective cohort study. Details on the rationale of the study, study design, sampling methods, response rate, and data collection have been published in detail previously^36,37^. In brief, 4814 participants aged 45–74 years were randomly selected between 2000 and 2003 from mandatory registries of residence of the cities Bochum, Essen and Mülheim/Ruhr within the densely populated Ruhr metropolitan area in Germany. All participants gave written informed consent. The study has been approved by the institutional ethics committee of the University Duisburg-Essen and was conducted according to the guidelines and recommendations for ensuring Good Epidemiological Practice^38^. An extended quality management procedure and certification according to DIN ISO 9001:2000 was established. All study participants were of European ancestry and thus genetically very homogeneous.

### Data collection

At study baseline, standard enzymatic methods (homogeneous direct determination with OPERA measuring system) were used to measure HDL-C, LDL-C and TC within 12 h after blood serum collection at the central laboratory of the University Hospital of Essen, Germany. Participants were asked to fast for at least 4 h before examination resulting in 60% of subjects with fasting status > 8 h, 2% 6–8 h, 5% 4–6 h, 26% 2–4 h and 7% with < 2 h of fasting. The fasting duration was on average 9.7 h (SD: ± 4.9 h), with no difference between men and women^39^. Information on educational attainment, household income, smoking status, physical activity and diagnosis of diabetes mellitus or use of anti-diabetic medication was collected at study baseline in standardized computer-assisted face-to-face interviews. Information on alcohol consumption was collected with a self-administered questionnaire. Education was defined by combining school and vocational training as total years of formal education according to the International Standard Classification of Education (ISCED 97)^40^. Years of education were categorized into three groups with ≤ 10 years (equivalent to a minimum compulsory school attendance with no additional vocational degree), 11–13 years (equivalent to upper secondary educational degrees or a combination of lower secondary education and vocational training), and ≥ 14 years of education (equivalent to a vocational training including additional qualification or a university degree). Income was measured as the monthly household equivalent income calculated by dividing the participants’ household net income by a weighting factor for each household member^41^. Income was categorized into three groups using sex-specific tertiles. In order to take account for their different mechanisms in causing health inequalities, both SEP indicators were analyzed separately^42,43^. The body-mass-index (BMI) was calculated based on standardized measurements of body weight (in underwear) and height (kg/m^2^). Physical activity was defined as exercising one and more times per week versus no weekly engagement in physical exercise. Smoking status was dichotomized for analyses as current smoker (smoking cigarettes during the past 12 months) versus former and never smoker. Alcohol intake was estimated as gram of pure alcohol per week using information on the number of alcoholic drinks usually consumed in a week by type of drink (i.e., beer, wine, sparkling wine, spirits). Participants were classified as diabetics if they reported a diagnosis of diabetes mellitus, or if a fasting blood glucose level ≥ 126 mg/dl, a postprandial blood glucose level ≥ 200 mg/dl was found, or if the use of anti-diabetic medication was documented. All variables have been checked in an ongoing data quality control during the baseline examination period.

### Genetic data

Lymphocyte DNA was isolated from EDTA anti-coagulated venous blood using the Chemagic Magnetic Separation Module I (Chemagen, Baesweiler, Germany). Genotyping was performed by matrix-assisted laser desorption ionization-time of flight mass spectrometry-based iPLEX Gold assay at the Department of Genomics, Life and Brain Center, Bonn Germany using two different Illumina microarrays (Metabochip, Global Screening Array (GSA); Illumina, San Diego, USA) according to the manufacturer’s protocols. Genotype imputation was carried out using IMPUTE v.2.3.0^44^. Quality control was applied prior to imputation and performed on subject level including sex-, ethnicity- and relatedness-checks, excluding subjects with missing genotype data > 10%. Further, single nucleotide polymorphisms (SNPs) with a missing genotype frequency > 10% were excluded.

Using the GLGC GWAS meta-analyses^14^ and all afterwards published large-scale GWAS^15–17^, 152 HDL-C-, 108 LDL-C- and 131 TC-associated SNPs at genome-wide significance level of *p* < 5 × 10^–8^ in study populations of European ancestry have been selected for analysis (Supplementary Table S1). Of these, 102, 85, and 103 genotyped SNPs or a proxy were found for HDL-C, LDL-C and TC, respectively, on the Metabochip. Additionally, 3/2/3 HDL-C/LDL-C/TC SNPs were found in imputed data of the Metabochip and 18/8/11 HDL-C/LDL-C/TC SNPs or a proxy were found on the GSA (Supplementary Table S1–S4). A proxy was defined as a SNP within a linkage disequilibrium (LD) ≥ 0.8. For all SNPs included in the analysis, no deviation from Hardy–Weinberg equilibrium was found (*p* ≤ 1 × 10^–6^). LD-based SNP pruning was performed with PLINK to exclude selected SNPs correlated with an LD ≥ 0.8 before calculating the GES_Lipid_. However, all SNPs included represented independent loci and none of the SNPs had to be pruned out. Two different GES_Lipid_ were calculated for each lipid trait: GES_Lipid_ based on^14^ and an extended GES_Lipid-EXT_ based on^14–17^. In detail, the genetic sum scores of effect alleles for HDL-C (GES_HDL-C_), LDL-C (GES_LDL-C_) and TC (GES_TC_) were calculated by aggregating the total number of lipid-increasing effect alleles (0,1 or 2) for each individual from the Heinz Nixdorf Recall Study population across the selected SNPs based on the GLGC GWAS meta-analyses^14^. For comparison, extended GES_Lipid-EXT_ have been calculated for each trait by additionally adding the number of lipid-increasing effect alleles of selected SNPs based on all afterwards published large-scale GWAS (i.e., GES_HDL-C-EXT_, GES_LDL-C-EXT_, GES_TC-EXT_)^15–17^. Imputation of missing genotype information was based on the study sample’s effect allele frequencies according to the PLINK scoring routine^45^.

### Statistical analyses

Out of the study population (n = 4814) all participants without genetic information (n = 296) and missing values for all three lipids (HDL-C, LDL-C and TC) (n = 22) were excluded from the analysis, leading to an analysis population of 4516 study participants (50.0% women) (Supplementary Figure S1). Participants with missing information on education (n = 13) and income (n = 283), were excluded from respective analysis. Compared to the analysis population, participants with missing genetic information as well as missing values in lipids and SEP indicators did not differ substantially regarding the main variables included in the analysis.

First, sex- and age-adjusted linear regression models were fitted to calculate effect size estimates and their corresponding 95% confidence intervals (95% CIs) for the association of education, income and the respective GES_Lipid_ (and GES_Lipid-EXT_) with each lipid trait. The explained variance of GES_Lipid_ (and GES_Lipid-EXT_) on lipids was calculated with a non-adjusted linear regression model. Second, the GES_Lipid_ (and GES_Lipid-EXT_) and SEP main effects as well as GES×SEP interaction terms were included into sex- and age-adjusted linear regression models to investigate GES×SEP interactions. Third, the genetic effect of GES_Lipid_ (and GES_Lipid-EXT_) on each lipid trait was calculated stratified by education groups, income tertiles and diabetes status. Fourth, all possible combinations of GES_Lipid_ (and GES_Lipid-EXT_) tertiles and SEP groups were entered into regression models as dummy variables to calculate single reference joint effects of the GES and the SEP indicators, using the group with the highest SEP and the lowest GES_LDL-C/TC_ tertile and accordingly the highest SEP and the highest GES_HDL-C_ tertile as reference. Additionally, absolute measures of lipids in each of the different combinations of SEP groups and GES_Lipid_ tertiles were calculated. Fifth, to analyze whether GES×SEP interactions may be affected by underlying interactions between GES_Lipid_ and SEP-related life style risk factors, smoking (S), BMI, physical activity (PA), alcohol consumption (A) or diabetes mellitus (D) main effects and the respective GES×S/BMI/PA/A/D interaction terms in addition to an SEP×S/BMI/PA/A/D interaction term were included in the interaction model separately for each life style risk factor. Single SNP main effect and single SNP interaction analysis between SNPs and education groups were performed for all SNPs used in the GES_Lipid_. Education is entered as a dummy variable and only the results of the lowest education group compared to the highest education group as reference were presented. Additionally, participants with lipid-lowering medication (n = 557) were considered in sensitivity analyses by (1) adjusting the main results of GES_Lipid_, education and GES×Education for lipid-lowering medication and by (2) excluding individuals with lipid-lowering medication. The LD-based pruning and the calculation of GES and single SNP analyses were performed with using PLINK v1.07 software package^45^ and RStudio v3.6.0^46^. For all other analyses SAS software v9.4^47^ was used. All GES_Lipid_- and extended GES_Lipid-EXT_-related beta coefficients and 95% CIs (except for the single reference joint effect analysis) were standardized by multiplying the coefficients by the standard deviation of the respective GES_Lipid_ or the respective extended GES_Lipid-EXT_ to facilitate comparability of each GES.

## Results

In the analysis population, the mean age (± standard deviation) was 59.6 ± 7.8 years and the mean serum lipid concentration (± standard deviation) were 58.16 ± 17.29 mg/dl for HDL-C, 145.41 ± 36.21 mg/dl for LDL-C and 229.26 ± 39.18 mg/dl for TC (Table 1). 11.4% had less than or equal 10 years of education and 33.0% had more than or equal 14 years of education. The median income was 1448.7 Euro/month. The mean number of effect alleles were 78.3 ± 5.1 for the GES_HDL-C_ (GES_HDL-C-EXT_: 136.4 ± 6.6), 55.1 ± 4.5 for the GES_LDL-C_ (GES_LDL-C-EXT_: 93.6 ± 5.9) and 75.7 ± 5.2 for the GES_TC_ (GES_TC-EXT_: 126.2 ± 6.6). 615 (13.6%) participants had diabetes mellitus. Correlation matrix of lipid phenotypes, GES_Lipid_, education and income is shown in Supplementary Table S5.

**Table 1:** Characteristics of analysis population.

	All (n = 4516)
Age (years)* [n_miss_ = 0]	59.6 ± 7.8
Female Sex^#^	2256 (50.0%)
Serum lipid concentration (mg/dl)*	
HDL-C [n_miss_ = 1]	58.16 ± 17.29
LDL-C [n_miss_ = 14]	145.41 ± 36.21
TC [n_miss_ = 0]	229.26 ± 39.18
Lipid-lowering medication [n_miss_ = 289]^#^	557 (13.2%)
**Number of lipid effect alleles (GES**_**Lipid**_^**§**^)*	
GES_HDL-C_ [n_miss_ = 0]	78.3 ± 5.1
GES_LDL-C_ [n_miss_ = 0]	55.1 ± 4.5
GES_TC_ [n_miss_ = 0]	75.7 ± 5.2
**Number of lipid effect alleles (GES**_**Lipid-EXT**_^**+**^)*	
GES_HDL-C-EXT_ [n_miss_ = 0]	136.4 ± 6.6
GES_LDL-C-EXT_ [n_miss_ = 0]	93.6 ± 5.9
GES_TC-EXT_ [n_miss_ = 0]	126.2 ± 6.6
**Education (years of training)**^**#**^ [n_miss_ = 13]	
≤ 10	515 (11.4%)
11–13	2502 (55.6%)
≥ 14	1486 (33.0%)
Income (€/month) † [n_miss_ = 283]	1448.7 (1107.8–1874.7)
Body mass index (kg/m2)* [n_miss_ = 25]	27.92 ± 4.64
No physical activity^#^	2302 (51.0%)
Current smoking^#^ [n_miss_ = 7]	1069 (23.7%)
Alcohol consumption† (g/week) [n_miss_ = 112]	13.9 (0–64.5)
Diabetes mellitus ^#^	615 (13.6%)

n_miss_ = number of participants with missing values, HDL-C = high-density lipoprotein cholesterol, LDL-C = low-density lipoprotein cholesterol, TC = total cholesterol, GES = genetic effect allele sum score, diabetes mellitus is defined as self-reported diabetes mellitus, or fasting blood glucose level ≥ 126 mg/dl or postprandial blood glucose level ≥ 200 mg/dl or if the use of anti-diabetic medication was documented.

*Mean ± standard deviation (SD)

^#^Proportion (%)

^†^Median (first quartile-third quartile)

^§^GES_Lipid_ based on^14^

^+^GES_Lipid-EXT_ based on^14–17^

SEP inequalities in HDL-C and LDL-C were found in the study population with worse HDL-C and LDL-C profiles observed in lower income and education groups (Table 2). SEP inequalities in TC were not seen. Participants in the lowest education group (≤ 10 years) had a 4.14 (95%-CI: -5.82, −2.47) mg/dl lower HDL-C, a 4.23 (95%-CI: 0.37, 8.09) mg/dl higher LDL-C and 3.34 (95%-CI: −0.81, 7.49) mg/dl higher TC level compared to the participants in the highest education group (≥ 14 years) with similar patterns for income (Table 2). On average, a 2.91 (95%-CI: (2.45, 3.37)) mg/dl higher HDL-C, a 6.17 (95%-CI: 5.13, 7.20) mg/dl higher LDL-C and a 7.33 (95%-CI: 6.24, 8.48) mg/dl higher TC were seen per standard deviation of the respective GES_Lipid_ (Table 2). The explained variance (R^2^) of GES_HDL-C_/ GES_LDL-C_/ GES_TC_ on their respective lipid was 2.9/ 2.9/ 3.6%.

**Table 2:** Sex- and age- adjusted effects per GES_Lipid_ standard deviation and corresponding 95% confidence intervals (95% CI) on high-density lipoprotein cholesterol (HDL-C), low-density lipoprotein cholesterol (LDL-C) and total cholesterol (TC) in linear regression models including main effects of education groups (≤ 10 years/11–13 years/≥ 14 years), income tertiles and genetic effect allele sum scores (GES_Lipid_) based on^14^.

	HDL-C		LDL-C		TC	
	β (95%-CI)	*p*	β (95%-CI)	*p*	β (95%-CI)	*p*
**Lipid ~ Education + age + sex**						
n	4502		4489		4503	
Intercept	34.28 (30.40; 38.15)	2.85*10^–65^	129.13 (120.22; 138.05)	2.3*10^–163^	196.18 (186.60; 205.76)	1.2*10^–301^
Age	0.05 (−0.01; 0.11)	0.092	0.26 (0.12; 0.39)	2.9*10^–04^	0.34 (0.19; 0.49)	6.3*10^–06^
Sex	14.85 (13.87; 15.82)	5.0*10^–179^	−0.72 (−2.96; 1.52)	0.530	7.75 (5.34; 10.16)	3.1*10^–10^
Education (low)	−4.14 (−5.82; −2.47)	1.4*10^–06^	4.23 (0.37; 8.09)	0.032	3.34 (−0.81; 7.49)	0.115
Education	−1.77 (−2.83; −0.72)	9.6*10^–04^	2.95 (0.53; 5.37)	0.017	1.21 (−1.39; 3.81)	0.360
Education (high)	Ref	–	Ref	–	Ref	–
**Lipid ~ Income + age + sex**						
n	4232		4220		4233	
Intercept	36.17 (32.24; 40.10)	3.1*10^–70^	127.00 (118.02; 136.00)	1.9*10^–155^	194.28 ( 184.68; 203.88)	7.6*10^–293^
Age	0.03 (−0.03; 0.09)	0.380	0.27 (0.13; 0.41)	1.9*10^–04^	0.36 (0.21; 0.51)	2.4*10^–06^
Sex	14.11 (13.16; 15.07)	6.5*10^–169^	0.01 (−2.17; 2.19)	0.995	7.95 (5.62; 10.29)	2.5*10^–11^
Income (low)	−1.09 (−2.24; −0.05)	0.061	3.91 (1.29; 6.53)	3.4*10^–03^	2.62 (−0.17; 5.42)	0.066
Income	−1.55 (−2.75; −0.34)	0.012	2.71 (−0.05; 5.46)	0.054	1.13 (−1.82; 4.07)	0.452
Income (high)	Ref	–	Ref	–	Ref	–
**Lipid ~ GES**_**Lipid**_** + age + sex**						
n	4515		4502		4516	
Intercept	−8.42 (−16.33; −0.51)	0.037	53.76 (38.51; 69.01)	5.6*10^–12^	89.74 (71.21; 108.26)	3.4*10^–21^
Age	0.02 (−0.04; 0.08)	0.533	0.27 (0.14; 0.41)	6.4*10^–05^	0.35 (0.21; 0.49)	1.8*10^–06^
Sex	14.05 (13.15; 15.00)	6.2*10^–185^	0.06 (−2.02; 2.14)	0.956	8.00 (5.77; 10.23)	2.2*10^–12^
GES_Lipid_	2.91 (2.45; 3.37)	7.5*10^–35^	6.17 (5.13; 7.20)	7.1*10^–31^	7.33 (6.24; 8.48)	2.0*10^–37^
**Lipid ~ GES**_**Lipid**_ **+ Education + age + sex**						
n	4502		4489		4503	
Intercept	−9.92 (−17.85; −1.98)	0.014	54.40 (39.06; 69.75)	4.2*10^–12^	90.80 (72.16; 109.44)	2.1*10^–21^
Age	0.05 (−0.01; 0.11)	0.110	0.25 (0.11; 0.38)	4.0*10^–04^	0.33 (0.18; 0.48)	9.4*10^–06^
Sex	14.79 (13.83; 15.75)	3.0*10^–183^	−1.03 (−3.23; 1.18)	0.363	7.35 (4.98; 9.71)	1.3*10^–09^
GES_Lipid_	2.91 (2.45; 3.36)	5.1*10^–35^	6.17 (5.13; 7.20)	6.9*10^–31^	7.33 (6.19; 8.42)	4.5*10^–37^
Education (low)	−4.12 (−5.77; −2.47)	1.0*10^–06^	4.73 (0.92; 8.53)	0.015	3.52 (−0.55; 7.60)	0.090
Education	−1.81 (−2.84; −0.77)	6.2*10^–04^	3.41 (1.03; 5.79)	5.1*10^–03^	1.49 (−1.06; 4.05)	0.252
Education (high)	Ref	–	Ref	–	Ref	–
**Lipid ~ GES**_**Lipid**_ **+ Income + age + sex**						
n	4232		4220		4233	
Intercept	−7.34 (−15.49; 0.82)	0.078	56.39 (40.66; 72.12)	2.4*10^–12^	92.54 (73.60; 111.48)	1.6*10^–21^
Age	0.02 (−0.04; 0.08)	0.480	0.27 (0.13; 0.40)	1.8*10^–04^	0.35 (0.20; 0.50)	3.0*10^–06^
Sex	14.08 (13.14; 15.01)	4.3*10^–173^	−0.14 (−2.30; 2.01)	0.895	7.67 (5.38; 9.96)	6.1*10^–11^
GES_Lipid_	2.86 (2.40; 3.32)	5.2*10^–32^	5.81 (4.73; 6.89)	3.5*10^–26^	7.07 (5.93; 8.22)	2.1*10^–33^
Income (low)	−1.08 (−2.20; 0.05)	0.061	3.57 (0.98; 6.15)	6.8*10^–03^	2.19 (−0.56; 4.94)	0.119
Income	−1.49 (−2.68; −0.31)	0.014	2.29 (−0.43, 5.01)	0.098	0.73 (−2.17; 3.62)	0.622
Income (high)	Ref		Ref		Ref	

In the linear regression analysis including interaction terms, effect size estimates of interaction terms showed stronger indication of GES_Lipid_ by low education interaction for LDL-C (ß_GES×Education_: −3.87; 95%-CI: −7.47, −0.32) compared to TC (ß_GES×Education_: −3.64; 95%-CI: −7.44; 0.16) and HDL-C (ß_GES×Education_: −0.56; 95%-CI: −2.09; 1.02) using the highest education group as reference (Table 3). The negative interaction coefficient showed that in the lower education group genetic effects of GES_Lipid_ were less strong. The effect size estimates for the GES_Lipid_ by income interaction were directionally consistent, except for TC, but substantially smaller in magnitude.

**Table 3:** Sex- and age- adjusted effects per GES_Lipid_ standard deviation and corresponding 95% confidence intervals (95% CI) on high-density lipoprotein cholesterol (HDL-C), low-density lipoprotein cholesterol (LDL-C) and total cholesterol (TC) in linear regression models including main effects of a lipid-associated genetic effect allele sum score (GES_Lipid_ based on^14^), indicators of socioeconomic position (SEP; education groups and income tertiles) and interaction terms of GES_Lipid_ and indicators of SEP.

	HDL-C		LDL-C		TC	
	β (95%-CI)	*p*	β (95%-CI)	*p*	β (95%-CI)	*p*
**Lipid ~ age + sex + GES**_**Lipid**_ **+ Education + GES**_Lipid_*Education						
n	4502		4489		4503	
Intercept	−11.50 (−23.95; 1.00)	0.070	49.89 (25.51; 74.26)	6.1*10^–05^	80.32 (50.25; 110.40)	1.7*10^–07^
Age	0.05 (−0.01; 0.11)	0.106	0.25 (0.11; 0.38)	4.1*10^–04^	0.33 (0.19; 0.478)	8.1*10^–06^
Sex	14.79 (13.83; 15.75)	3.9*10^–183^	−1.07 (−3.28; 1.14)	0.341	7.35 (5.00; 9.72)	1.2*10^–09^
GES_Lipid_	3.01 (2.24; 3.77)	5.2*10^–14^	6.53 (4.68; 8.37)	5.2*10^–12^	8.01 (6.08; 9.98)	1.2*10^–15^
Education (low)	4.33 (−19.59; 28.26)	0.723	52.31 (8.39; 96.24)	0.020	56.57 (0.93; 112.21)	0.046
Education	−0.68 (−16.00; 14.64)	0.931	1.84 (−26.52; 30.20)	0.899	8.88 (−27.09; 44.86)	0.628
Education (high)	Ref	–	Ref	–	Ref	–
GES_Lipid_ × Edu^#^ (low)	−0.56 (−2.09; 1.02)	0.487	−3.87 (−7.47; −0.32)	0.033	−3.64 (−7.44; 0.16)	0.062
GES_Lipid_ × Edu^#^	−0.05 (−1.07; 0.92)	0.885	0.14 (−2.16; 2.43)	0.911	−0.52 (−2.96; 1.98)	0.686
GES_Lipid_ × Edu^#^ (high)	Ref	–	Ref	–	Ref	–
**Lipid ~ age + sex + GES**_**Lipid**_ **+ Income + GES**_Lipid_*Income						
n	4232		4220		4233	
Intercept	−8.60 (−21.34; 4.14)	0.186	55.82 (31.47; 80.16)	7.2*10^–06^	88.25 (58.43; 118.07)	7.0*10^–09^
Age	0.02 (−0.04; 0.08)	0.483	0.27 (0.13; 0.40)	1.8*10^–04^	0.35 (0.20; 0.50)	3.2*10^–06^
Sex	14.07 (13.14; 15.01)	5.5*10^–173^	−0.14 (−2.41; 1.90)	0.897	7.66 (5.37; 9.95)	6.5*10^–11^
GES_Lipid_	2.96 (2.14; 3.72)	5.4*10^–13^	5.85 ( 4.01; 7.70)	6.8*10^–10^	7.38 ( 5.46; 9.31)	1.2*10^–13^
Income (low)	0.80 (−16.72; 18.33)	0.928	6.54 (−24.83; 37.91)	0.683	−4.95 (−44.85; 34.94)	0.808
Income	0.56 (−17.31; 18.42)	0.951	0.40 (−32.81; 33.61)	0.981	23.38 (−18.03; 64.79)	0.269
Income (high)	Ref	–	Ref	–	Ref	–
GES_Lipid_ × Inc^$^ (low)	−0.10 (−1.28; 1.02)	0.833	−0.23 (−2.79; 2.30)	0.852	0.47 (−2.24; 3.22)	0.726
GES_Lipid_ × Inc^$^	−0.15 (−1.28; 1.02)	0.822	0.14 (−2.57; 2.84)	0.911	−1.56 (−4.42; 1.30)	0.283
GES_Lipid_ × Inc^$^ (high)	Ref	–	Ref	–	Ref	–

^#^Education, ^$^Income.

**Figure 1:**
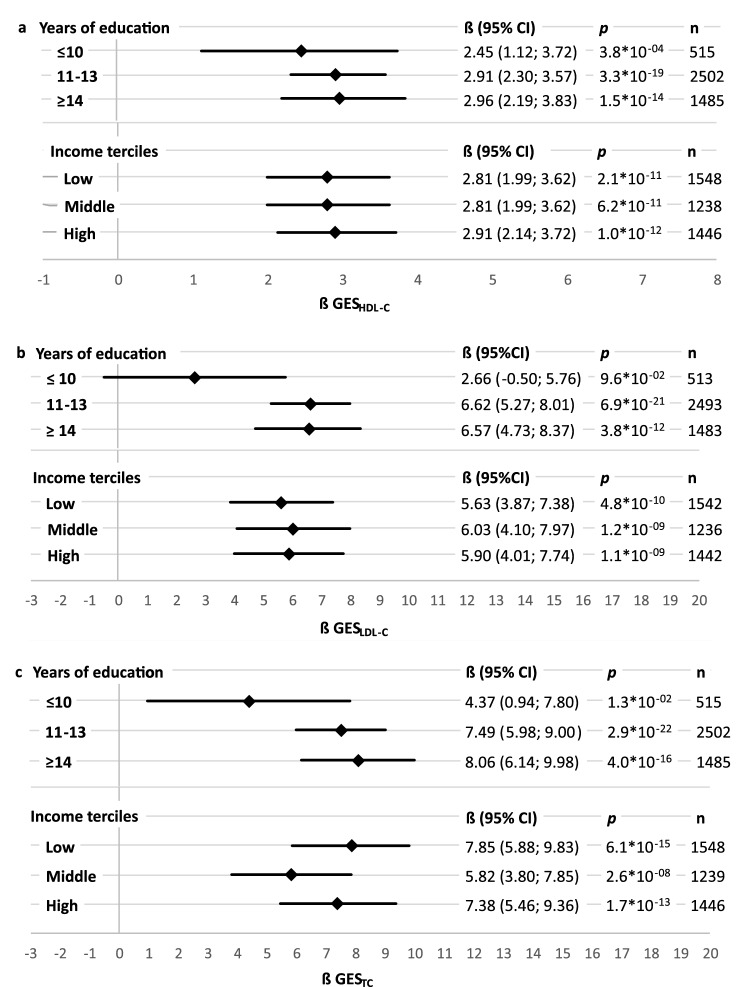
Sex-, age-adjusted effects per GES_Lipid_ standard deviation and corresponding 95% confidence interval (95% CI) of the genetic effect on (**a**) high-density lipoprotein cholesterol (HDL-C), (**b**) low-density lipoprotein cholesterol (LDL-C) and (**c**) total cholesterol (TC), stratified by education groups (years) and income tertiles in linear regression models using the GES_Lipid_ based on^14^.

In the stratified analysis, the two higher education groups compared to the lowest showed stronger genetic effect size estimates per GES_Lipid_ standard deviation for LDL-C and TC, supporting the results of the interaction analysis (Fig. 1). The results for HDL-C followed the same pattern, but the difference in effect size between the highest and the lowest education group was considerably less strong, while the 95% confidence interval of the effect in the lowest education group was completely overlapping with the 95% confidence intervals of both higher education groups. The results of the stratified analysis for income did not follow a clear pattern (Fig. 1). The partial R^2^ (explained proportion of variance) of the GES_LDL-C_ on LDL-C and GES_TC_ on TC in the respective education groups was higher in the two higher education groups (LDL-C: high education group R^2^ = 0.033, middle education group R^2^ = 0.035; TC: high education group R^2^ = 0.044, middle education group R^2^ = 0.038) compared to the lower education group (LDL-C: R^2^ = 0.005; TC: R^2^ = 0.013). For Income and HDL-C this trend could not be observed.

**Figure 2:**
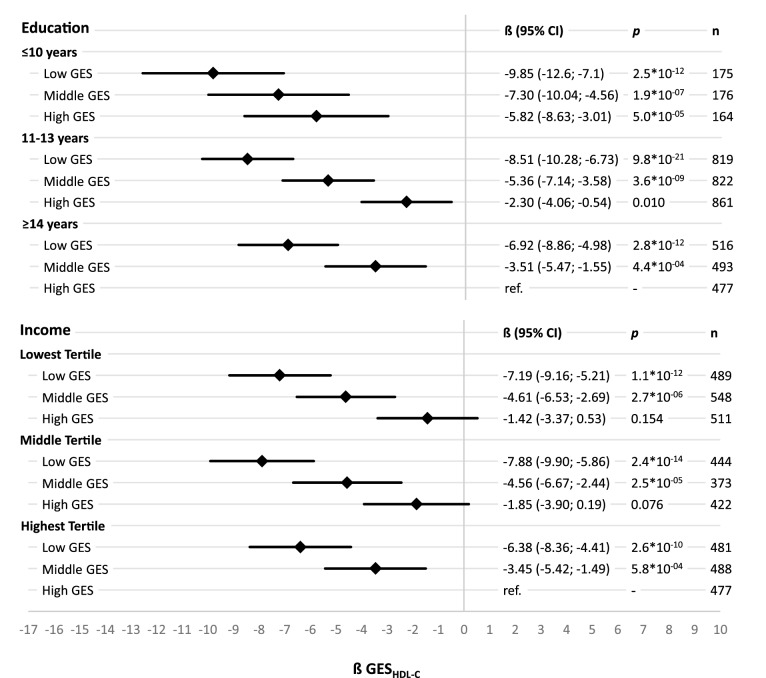
Sex- and age-adjusted effects and corresponding 95% confidence intervals (95% CI) on high-density lipoprotein cholesterol (HDL-C) in linear regression models for single reference joint effects of tertiles of a HDL-C-associated genetic effect allele sum score (GES_HDL-C_ based on^14^) and socioeconomic position indicators, calculated separately for education groups and income tertiles, with the group of having a high GES_HDL-C_ and the highest socioeconomic position as reference.

**Figure 3:**
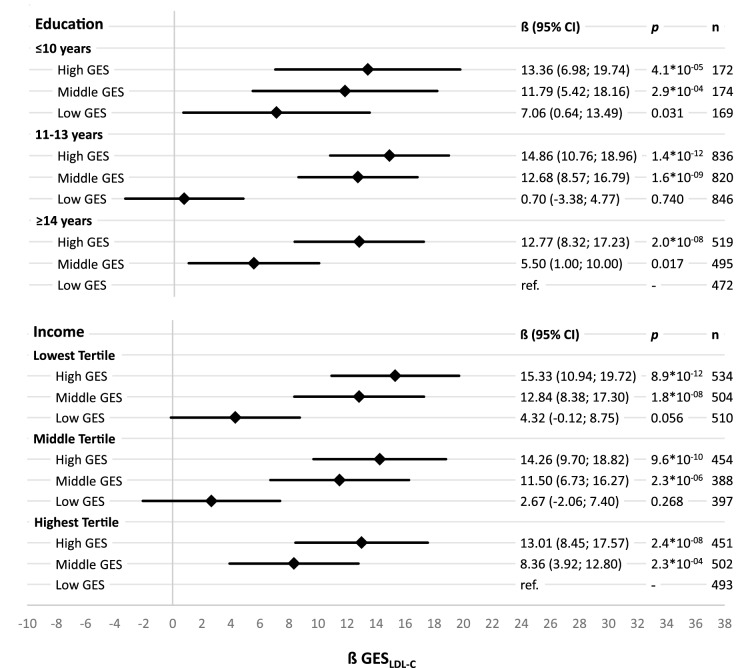
Sex- and age-adjusted effects and corresponding 95% confidence intervals (95% CI) on low-density lipoprotein cholesterol (LDL-C) in linear regression models for single reference joint effects of tertiles of a LDL-C-associated genetic effect allele sum score (GES_LDL-C_ based on^14^) and socioeconomic position indicators, calculated separately for education groups and income tertiles, with the group of having a low GES_LDL-C_ and the highest socioeconomic position as reference.

**Figure 4:**
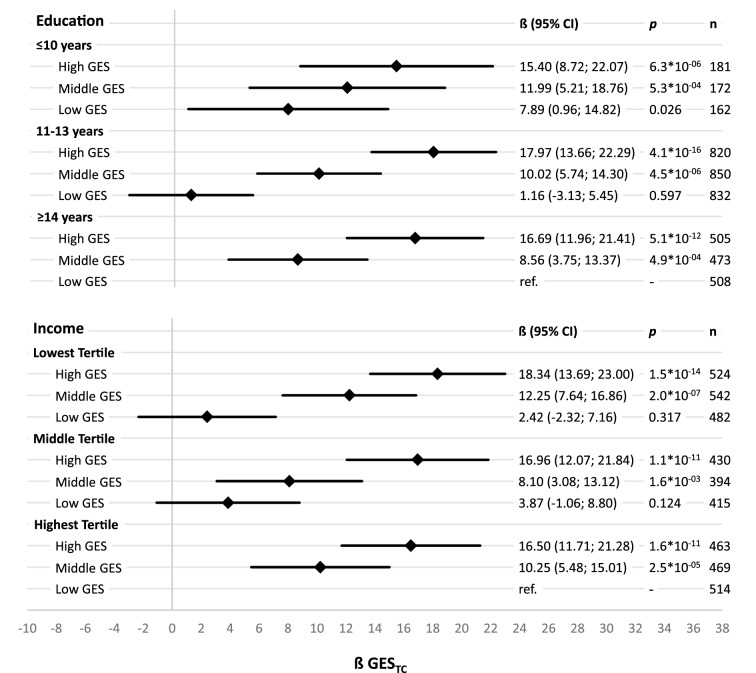
Sex- and age-adjusted effects and corresponding 95% confidence intervals (95% CI) on total cholesterol (TC) in linear regression models for single reference joint effects of tertiles of a TC-associated genetic effect allele sum score (GES_TC_ based on^14^) and socioeconomic position indicators, calculated separately for education groups and income tertiles, with the group of having a low GES_TC_ and the highest socioeconomic position as reference.

The analysis of single reference joint effects for lipids describe the relationship between SEP and GES_Lipid_ on lipids in detail by comparing effects of different combinations of SEP groups and GES_Lipid_ tertiles. Each beta estimate represent the increase in lipids of the specific group compared to the reference group. Reference group was selected as the combination of GES_LDL-C_ and SEP group representing the lowest CVD risk (equally applied for GES_TC_ and GES_HDL-C_). For HDL-C beta estimates showed a downward trend between and within education groups with decreasing years of education and decreasing number of effect alleles. Compared to the reference group with the highest education and highest GES_HDL-C_, participants with the lowest education and the lowest GES_HDL-C_ showed a 9.85 mg/dl lower HDL-C level. Slightly smaller joint effects were observed for income and GES_HDL-C_ on HDL-C still following the same pattern (Fig. 2). The joint effects for LDL-C and TC showed an upward trend with decreasing years of education and increasing number of effect alleles. Participants with highest CVD-risk (highest GES_Lipid_ and lowest education) had a 13.36 mg/dl higher LDL-C and a 15.40 mg/dl higher TC level than those with the lowest CVD-risk (highest education and lowest GES_Lipid_). Slightly stronger joint effects were observed for income and GES_Lipid_ on LDL-C and TC (Figs. 3, 4). The absolute measures of lipids in each of the different combinations of SEP groups and GES_Lipid_ tertiles show the same pattern as in the single reference joint effect analysis (Supplementary Figures S3–S5). Participants in the highest GES_Lipid_ tertile and the lowest education had on average 8.8/14.0/21.1 mg/dl higher HDL-C/LDL-C/TC level than participants in the lowest GES_Lipid_ tertiles and the highest education. Almost similar measures have been observed for income and GES_Lipid_ tertiles.

After including interaction terms of smoking, BMI, physical activity, alcohol consumption and diabetes separately into the GES_LDL-C_×Education interaction model, we observed a GES_LDL-C_×Diabetes interaction effect (ß_GES×Diabetes_: −4.46; 95%-CI: −7.38, −1.53) indicating less strong genetic effects on LDL-C in diabetics compared to non-diabetics (Table 4), which was also observed in the stratified analysis (Supplementary Figure S6). Including the GES_LDL-C_×Diabetes interaction effect also partly explained the GES_LDL-C_×Education interaction effect of the lowest compared to the highest education group, as the effect estimate was attenuated (ß_GES×Education_: −3.42; 95%-CI: −6.98, 0.18) (Table 4). The GES_LDL-C_×Education interaction was not affected by other life style risk factors, as the respective GES_LDL-C_×low education interaction effect size estimates did not change in magnitude after including smoking, BMI, physical activity and alcohol consumption in the regression models (Table 4). In addition, results did not indicate GES_LDL-C_ by life style risk factor interactions.

**Table 4:** Sex- and age- adjusted effects per GES_LDL-C_ standard deviation and corresponding 95% confidence intervals (95% CI) on LDL-C in linear regression models including main effects and interaction terms of a LDL-C-associated genetic effect allele score (GES_LDL-C_ based on^14^), education groups and SEP-related life style risk factors (i.e., current smoking [S], BMI, physical activity [PA], alcohol consumption [A; per 100 g/week]) and diabetes mellitus [D].

	LDL-C ~ age + sex + Edu + GES_LDL-C_ + S + GES_LDL-C_*Edu + S*Edu + GES_LDL-C_*S		LDL-C ~ age + sex + Edu + GES_LDL-C_ + BMI + GES_LDL-C_*Edu + BMI *Edu + GES_LDL-C_* BMI		LDL-C ~ age + sex + Edu + GES_LDL-C_ + PA + GES_LDL-C_*Edu + PA*Edu + GES_LDL-C_*PA		LDL-C ~ age + sex + Edu + GES_LDL-C_ + A + GES_LDL-C_*Edu + A*Edu + GES_LDL-C_*A		LDL-C ~ age + sex + Edu + GES_LDL-C_ + D + GES_LDL-C_*Edu + D*Edu + GES_LDL-C_*D	
Education	β (95%-CI)	*p*	β (95%-CI)	*p*	β (95%-CI)	*p*	β (95%-CI)	*p*	β (95%-CI)	*p*
n	4489		4466		4489		4382		4489	
Intercept	41.87 (16.61; 67.14)	1.2*10^–03^	−8.24 (−87.26; 70.77)	0.840	49.58 (23.44; 75.71)	2.0*10^–04^	50.37 (23.74; 77.00)	2.1*10^–04^	42.00 (17.20; 66.79)	9.1*10^–04^
Age	0.31 (0.17; 0.45)	1.1*10^–05^	0.21 (0.07; 0.35)	2.7*10^–03^	0.24 (0.11; 0.38)	4.9*10^–04^	0.25 (0.12; 0.39)	3.1*10^–04^	0.28 (0.14; 0.42)	5.4*10^–05^
Sex	−0.75 (−2.96; 1.47)	0.510	−0.36 (−2.59; 1.86)	0.750	−0.83 (−3.06; 1.39)	0.460	−1.33 (−3.70; 1.04)	0.270	−1.67 (−3.89; 0.55)	0.140
Edu^#^ (low)	54.93 (11.06; 98.81)	0.014	59.72 (11.21; 108.22)	0.016	54.62 (9.89; 99.34)	0.017	53.68 (8.38; 98.98)	0.020	47.41 (3.53; 91.30)	0.034
Edu^#^	4.06 (−24.37; 32.49)	0.780	9.15 (−22.63; 40.94)	0.570	2.29 (−26.48; 31.05)	0.880	2.94 (−25.90; 31.79)	0.840	0.92 (−27.38; 29.21)	0.949
Edu^#^ (high)	ref	–	ref	–	ref	–	ref	–	ref	–
GES_LDL-C_	6.71 (4.82; 8.64)	7.1*10^–12^	9.41 (3.06; 15.75)	3.8*10^–03^	6.48 (4.50; 8.51)	2.1*10^–10^	6.48 (4.41; 8.51)	5.7*10^–10^	7.16 (5.27; 9.05)	1.4*10^–13^
S	9.85 (−20.47; 40.16)	0.520	–	–	–	–	–	–	–	–
BMI	–		2.19 (–0.59; 4.97)	0.120	–	–	–	–	–	–
PA	–		–	–	−0.04 (−26.25; 26.16)	1.000	–	–	–	–
A	–		–	–	–	–	0.00 (−0.13; 0.14)	0.950	–	–
D									46.97 (10.46; 83.48)	0.012
GES_LDL-C_ × Edu^#^ (low)	−4.23 (−7.83; −0.68)	0.020	−3.83 (−7.43; −0.18)	0.040	−3.96 (−7.56; −0.32)	0.033	−3.87 (−7.56; −0.18)	0.040	−3.42 (−6.98; 0.18)	0.061
GES_LDL-C_ × Edu^#^	0.00 (−2.30; 2.30)	1.000	0.14 (−2.16; 2.48)	0.900	0.05 (−2.30; 2.39)	0.970	0.09 (−2.25; 2.43)	0.950	0.223 (−2.07; 2.52)	0.861
GES_LDL-C_ × Edu^#^ (high)	ref	–	ref	–	ref	–	ref	–	ref	–
GES_LDL-C_ × S	−0.26 (2.70; 2.21)	0.850	–	–	–	–	–	–	–	-
GES_LDL-C_ × BMI	–	–	−0.09 (−0.32; 0.14)	0.350	–	–	–	–	–	–
GES_LDL-C_ × PA	–	–	–	–	0.14 (−1.98; 2.25)	0.900	–	–	–	–
GES_LDL-C_ × A	–	–	–	–	–	–	0.00 (0.00; 0.00)	0.960	–	–
GES_LDL-C_ × D									−4.46 (−7.38; −1.53)	3.0*10^–03^
S × Edu^#^ (low)	6.78 (−2.07; 15.62)	0.130	–	–	–	–	–	–	–	–
S × Edu^#^	−4.51 (−10.02; 0.99)	0.110	–	–	–	–	–	–	–	–
S × Edu^#^ (high)	ref	–	–	–	–	–	–	–	–	–
BMI × Edu^#^ (low)	–	–	−0.34 (−1.06; 0.38)	0.350	–	–	–	–	–	–
BMI × Edu^#^	–	–	−0.30 (−0.82; 0.22)	0.260	–	–	–	–	–	–
BMI × Edu^#^ (high)	–	–	ref	–	–	–	–	–	–	-
PA × Edu^#^ (low)	–	–	–	–	−3.33 (−10.70; 4.04)	0.380	–	–	–	–
PA × Edu^#^	–	–	–	–	0.83 (−3.82; 5.48)	0.730	–	–	–	–
PA × Edu^#^ (high)	–	–	–	–	ref	–	–	–	–	–
A × Edu^#^ (low)	–	–	–	–	–	–	−0.04 (−0.08; 0.00)	0.057	–	–
A × Edu^#^	–	-	–	–	–	–	0.00 (−0.02; 0.02)	0.950	–	–
A × Edu^#^ (high)	–	–	–	–	–	–	ref	–	–	–
D × Edu^#^ (low)									−0.36 (−9.95; 9.23)	0.941
D × Edu^#^									1.52 (−5.31; 8.34)	0.663
D × Edu^#^ (high)									ref	–

^#^Education

Results of the extended GES_Lipid-EXT_, which included additional SNPs selected from recent large-scale GWAS, showed overall smaller effect size estimate per GES_Lipid-EXT_ standard deviation for all three lipid traits compared to the GES_Lipid_ (Supplementary Table S6). The explained variance (R^2^) of the extended GES_HDL-C-EXT_ was slightly higher (3.2%) compared to the GES_HDL-C_ and for the extended GES_LDL-C-EXT_ (2.6%) and GES_TC-EXT_ (3.4%) slightly lower compared to the GES_LDL-C_ and GES_TC_. In the extended GES_Lipid-EXT_ by SEP indicator interaction analysis, using the highest SEP groups as reference, effect size estimates of interaction terms were overall slightly smaller in magnitude for all three lipid levels compared to the GES_Lipid_ (Supplementary Table S7). Effects of the extended GES_Lipid-EXT_ stratified by education groups showed similar patterns for HDL-C, LDL-C, and TC compared to the GES_Lipid_. However, differences in the genetic effects between education groups were less strong in magnitude, while for income no difference in the genetic effects were observed (Supplementary Figure S2). The downward trend between and within education groups with decreasing years of education and decreasing number of effect alleles in the analysis of single reference joint effects for HDL-C and the upward trend for LDL-C and TC for both SEP indicators also showed the same pattern using the extended GES_Lipid-EXT_ compared to the GES_Lipid_ (Supplementary Tables S8–S9).

Results of the single SNP main effect of all SNPs used in the GES_Lipid_ are presented in Supplementary Tables S2–S4. 61 out of 71 HDL-C-, 52 out of 58 LDL-C-, 65 out of 74 TC-associated SNPs were directionally consistent. Single SNP interaction analysis for education and lipid-associated SNPs showed that some SNPs contributed with stronger effects to the observed GES_LDL-C_ by low education interaction on LDL-C in relation to the remaining SNPs (Supplementary Table S10). Similar differences of single SNP interaction effect size estimates were present for HDL-C- and TC-associated SNPs (Supplementary Tables S11–S12). Two of the LDL-C-associated loci with the strongest indication for interaction with education (i.e., *PCSK9*, *MAFB*) were also upon the strongest TC-associated SNPs. The two HDL-C-associated loci with the strongest indication for interaction with education were *OR4C46* and *LPL*.

In the sensitivity analysis, main results for all lipid traits did not differ in direction and only slightly in magnitude after adjustment for lipid-lowering medication as well as after the exclusion of participants on lipid-lowering medication (Supplementary Tables S13–S14). The GES_LDL-C_ by low education interaction effect size estimates did not change substantially compared to the main analysis population.

## Discussion

The aim of the study was to investigate whether the SEP indicators education and income interact with genetic sum scores of lipid-increasing effect alleles in a population-based cohort study. To the best of our knowledge, this was the first study investigating G×E interaction with SEP as environmental factor on lipids in adults. Results gave some indication for an interaction between the GES_Lipid_ and the SEP indicator education, which was strongest for LDL-C. This was supported by stratified analysis in which the strongest genetic effects on LDL-C were observed in the high education group as well as by single reference joint effect analysis. After including information on smoking, BMI, physical activity, alcohol consumption and diabetes mellitus into analysis, there was an indication that a GES_LDL-C_ by diabetes mellitus interaction partly explained the observed GES_LDL-C_ by low education interaction. Using the extended GES_Lipid-EXT_ in comparison to the GES_Lipid_, effect size measures were smaller but directionally consistent. Li et al. reported an interaction between rs2206734 SNP (*CDKAL1*), a favorable childhood environment and birthweight on metabolically obese, normal weight phenotype (defined as the presence of hypertension, hypertriglyceridemia, low serum HDL-C or impaired fasting plasma concentrations of glucose) in Chinese children. Their findings suggest that a favorable childhood environment represented by a composite score consisting of parental education, annual household income, high physical activity and fruit consumption can further amplify a protective effect of the *CDKAL1* locus in children with a pediatric metabolic syndrome and high birthweight^33^. However, this study investigated a composite environmental score with parental SEP on a composite children’s phenotype in Chinese and can therefore only indirectly be compared with present study results.

Recent studies have investigated statistical interactions between genetic risk scores and SEP-related lifestyle factors or health behaviors such as physical activity, dietary patterns and BMI and their effects on lipids^27,28^. Cole et al. (2014) have demonstrated in a population of European ancestry that the effect of a genetic risk score consisting of HDL-C-increasing alleles has been stronger for lean than for obese (BMI ≥ 35 kg/m^2^) study participants. These interactions have been largely driven by the SNPs rs3764261 (*CETP*), rs4846914 (*GALNT2*), rs7241918 (*LIPG*) and rs6065906 (*PLTP*)^28^. As high education is strongly associated with low BMI^48^, these results may at least partly reflect the results of the present study. However, in the present study the SNPs representing the loci *CETP*, *GALNT2*, *LIPG* and *PLTP* did not show indication for SNP by education interaction on HDL-C.

Justesen et al. (2015) have reported an interaction between a genetic risk score of HDL-C-decreasing effect alleles and physical activity in a Danish population (n = 5961), suggesting that the genetic risk score has exerted a smaller effect in physically active compared to inactive individuals. However, this interaction was statistically not significant in a replication cohort of smaller sample size^27^. As higher education is usually associated with a higher level of physical activity^49^, the results of the present study may represent the same interaction signal, i.e. a stronger genetic effect of HDL-C-increasing effect alleles on HDL-C in population groups of higher education groups.

Recent SNP×E interaction analyses have identified several lipid-associated loci interacting with lifestyle factors such as smoking and diet^50–52^. While Junyent et al. (2009) have reported an interaction of rs6720173 (*ABCG5*) and rs11887534, rs6709904, rs4148217 (*ABCG8*) with smoking^50^, Lu et al. (2010) have shown an interaction of rs174546 (*FADS1*) with intake of n-3 and n-6 polyunsaturated fatty acid^51^ and Kim et al. (2013) have demonstrated an interaction of rs2072183 (*NPC1L1*) with cholesterol intake in male only^52^. Although SEP is strongly associated with smoking and dietary factors, none of these loci showed indication for interaction with education in the present study. The GES_LDL-C_×Diabetes mellitus interaction on LDL-C observed in the present study partly explained the GES_LDL-C_×Education interaction. Deng et al. found a Gene×Diabetes mellitus interaction. In their study the SNP rs16996148 (*CILP2*) decreased the risk of hyperlipidemia, whereas rs16996148 GT/TT and diabetes mellitus as well as rs16996148 TT and diabetes mellitus increased the risk of hyperlipidemia^29^.

In the present analysis, smaller effects on lipids were observed for using the extended GES_Lipid-EXT_ compared to the GES_Lipid_. This may be caused by the overall smaller effect size of newly discovered loci as a result of larger analysis populations in recent GWAS, making it possible to detect risk alleles with very small effects. It may also be due to the recently published GWAS meta-analyses that were based on single large cohorts potentially producing less generalizable study results^16,17^. The overall smaller main effects of the additional SNPs included in the extended GES_Lipid-EXT_ have led to smaller interaction effect size estimates, i.e. less strong impact of SEP on the expression of the average genetic effect of all SNPs included.

It was assumed in the present analysis that the GES_Lipid_ represent cumulative causal factors for lipids even if it is most likely that the SNPs used to construct the GES_Lipid_ are proxy markers in high LD with the causal genetic variants^53^. The effect of SEP, especially education, on lipids and CVD risk in general was also assumed to be causal, as supported by numerous studies^54–56^ including mendelian randomization studies exploring the association of instrumental variables with CVD and CVD risk factors by using genetic risk scores related to educational attainment^57,58^. However, SEP has no direct causal effect on CVD risk, but is mediated by a complex interplay of social inequalities in risk factors, e.g., access to preventive interventions, lifestyle factors, physiological stress, psychosocial risks, as well as in protective factors^54,57,59^. Results of the present study suggest that SEP may also have an effect on CVD risk by affecting the expression of LDL-C-related genetic risks. One possible mechanism that has been hypothesized in this regard is epigenetic modification. In contrast to an individual’s genome the epigenome is subject to environmentally induced changes during the life course, but is crucial for the regulation of gene expression. Several studies have indeed reported SEP-related differences in epigenetic markers^60,61^. Interestingly, the lifestyle factors BMI, physical activity, smoking and alcohol consumption did not account for the observed GES_LDL-C_ by education interaction, while diabetes mellitus accounted for it only partly. Consequently, it has to be assumed that other risk factors besides those included in the present analysis may have a mediating effect on the found GES_LDL-C_ by education interaction. One explanation for the stronger genetic effects on LDL-C in higher education groups may be that non-genetic health risks being of lower prevalence in high education groups leading to LDL-C profiles that are stronger affected by genetic than by non-genetic risk factors. This hypothesis is supported by the explained proportion of the variance (R^2^) of the GES_LDL-C_ on LDL-C, which was higher in the two higher education groups compared to the lower education group.

The effect of SEP indicators on health is outcome specific and each indicator operates via different pathways linking social factors to health outcomes^43,62^. Even though educational attainment and income are moderately correlated (r = 0.45) in the present study, the different strength of genetic effect in education groups on LDL-C could not be seen in income tertiles. The net effect of education is reflected among others in the ability to turn health related information into behavior and facilitates understanding of therapeutic measures^43^. Which could support the hypothesis that in highly educated, due to the ability to create environments with less health risks, genetic influence on LDL-C might be stronger. Furthermore, education as a marker of childhood social environment could, due to the duration of exposure until adulthood, be more likely support epigenetic changes. Material resources do not seem to modify genetic risk on LDL-C.

Strengths of the present study were its population-based study sample and the use of two different individual SEP indicators in the analysis. Even though education and income are correlated SEP indicators, each of them represents certain aspects of SEP related to different health behaviors and risks. Moreover, two different GES_Lipid_ for each lipid trait were compared, allowing to check for differences in the genetic effects and G×E interactions between scores derived by different GWAS study populations. The sample size and the limited statistical power for single SNP analysis had to be mentioned as limitation of the present study. However, indication for interaction was based on the cumulative genetic risk of the study participants. Another limitation was the cross-sectional design of the study that does not allow for strong conclusions on causality of effects. However, educational attainment is usually acquired in adolescence or early adulthood and lipids were assessed at an older age in the present study. Due to this exposure-outcome temporality reverse causation is very unlikely. Even if the effect of education on lipids was not causal, a modification of the GES_LDL-C_ effect on LDL-C by education would still be of interest, because the knowledge of the heterogeneous genetic effects in different education groups could be interesting for CVD risk prediction and genotype-based targeted interventions^35^. Furthermore, this knowledge supports CVD lifestyle-based interventions in lower education groups due to lower genetic effect on LDL-C in these groups. Finally, it cannot be excluded that the indication for a GES_LDL-C_ × Education interaction could have been observed randomly due to the number of independent tests performed. However, the number of independent tests performed are justified through the three lipid end points and the two SEP indicators and we have calculated 95% confidence intervals to report the precision of the obtained effect size estimates. Furthermore, the interaction analyses results are supported by the results of the stratified and single reference joint effect analysis, which showed a constant pattern across phenotypes and indicated difference in genetic effect sizes between the education groups.

The results of the present study gave some indication for interaction between genetic variants associated with LDL-C and education in a population-based cohort study. Stronger genetic effects were observed in groups of higher education, which seemed to be partly mediated by diabetes mellitus but not by other life style risk factors such as BMI, smoking, physical activity and alcohol consumption. This gave supporting evidence that SEP has an impact on the expression of genetic susceptibility related to LDL-C. Further research is needed to replicate our findings in independent study samples, investigate possible biological mechanisms behind the interaction and to assess the potential of the found gene by SEP interactions for improving CVD prediction. Additionally, our study included only individuals of European origin and therefore the results may not be applied to populations of other ethnicities.

## Supplementary Information

Supplementary Information.

## Data Availability

Due to data security reasons (i.e., data contain potentially participant identifying information), the Heinz Nixdorf Recall Study does not allow sharing data as a public use file. However, other authors are allowed to access data upon request, which is the same way authors of the present paper obtained the data. Data requests can be addressed to: recall@uk-essen.de.
