# Supplementary material for: A genetic sum score of effect alleles associated with serum lipid concentrations interacts with educational attainment
Source: Sci Rep. 2021 Aug 16;11:16541. doi: 10.1038/s41598-021-95970-z (PMC8368036; doi:10.1038/s41598-021-95970-z)
Supplement: Supplementary file 1 — Supplementary Information. [file 41598_2021_95970_MOESM1_ESM.docx]

**A Genetic Sum Score of Effect Alleles Associated with Serum Lipid Concentrations Interacts with Educational Attainment**

Carina Emmel, M.Sc^*^; Mirjam Frank, PhD; Nico Dragano, PhD; Markus M. Nöthen, MD; Raimund Erbel, MD; Karl-Heinz Jöckel, PhD; Börge Schmidt, PhD

SUPPLEMENTARY MATERIAL

**Table S1:** Overview of the selection of lipid-associated single nucleotide polymorphisms (SNPs) used in the calculation of genetic effect allele sum scores (GES_Lipid_ and GES_Lipid-EXT_) in data of the Heinz Nixdorf Recall Study (HNR).

| **Lipid-associated single nucleotide polymorphisms (SNPs) in Genetic effect allele sum score (GES)** | **published SNPs** | **number of SNP included in GES** | **found in Metabochip** | **found in GSA chip** | **found in imputed Metabochip** | **SNPs not found and not included in GES**_Lipid_ |
| --- | --- | --- | --- | --- | --- | --- |
| HDL-C-associated SNPs in GES_Lipid_^§^ | 71 | 71 | 68 | 1 | 2 | 0 |
| HDL-C-associated SNPs in GES_Lipid-EXT_^#^ | 152 | 123 | 102 | 18 | 3 | 29 |
| LDL-C-associated SNPs in GES_Lipid_^§^ | 58 | 58 | 56 | 0 | 2 | 0 |
| LDL-C-associated SNPs in GES_Lipid-EXT_^#^ | 108 | 95 | 85 | 8 | 2 | 13 |
| TC-associated SNPs in GES_Lipid_^§^ | 74 | 74 | 72 | 0 | 2 | 0 |
| TC-associated SNPs in GES_Lipid-EXT_^#^ | 131 | 117 | 103 | 11 | 3 | 14 |
| ^§^Global Lipids Genetics Consortium (GLGC 2013) [14], ^#^GLGC 2013, Surakka et al. 2015, Hoffmann et al. 2018, Klarin et al. 2018 [14-17] | | | | | | |

**Table S2:** Overview of 71 loci associated with HDL-C included into genetic effect allele sum score GES_HDL-C_ based on [14] and 52 loci added to the 71 loci in the genetic effect allele sum score GES_HDL-C-EXT_ based on [15-17].

| **Chr:Position^1^** | **Original SNP** | **Proxy SNP^2^** | **Linkage disequi-librium^3^ (LD=r^2^)** | **Imputation quality** | **Effect allele/Non-effect allele** | **Effect allele frequency** | **Effect of effect allele** | **Lower 95%** | **Upper 95%** | **P value** |
| --- | --- | --- | --- | --- | --- | --- | --- | --- | --- | --- |
| **GES_HDL-C_** | | | |  |  |  |  |  |  |  |
| 1:156700651 | rs12145743 |  |  |  | G/T | 0.36 | 0.60 | -0.08 | 1.28 | 0.085 |
| 1:178515312 | rs4650994 |  |  |  | G/A | 0.47 | 0.29 | -0.35 | 0.94 | 0.373 |
| 1:182168885 | rs1689800 |  |  |  | A/G | 0.62 | 0.78 | 0.10 | 1.46 | 0.024 |
| 1:230295691 | rs4846914 |  |  |  | A/G | 0.61 | 0.50 | -0.17 | 1.16 | 0.141 |
| 1:27138393 | rs12748152 |  |  |  | C/T | 0.92 | 1.19 | -0.01 | 2.39 | 0.052 |
| 1:40028180 | rs4660293 |  |  |  | A/G | 0.75 | 0.22 | -0.53 | 0.97 | 0.572 |
| 2:165540800 | rs12328675 |  |  |  | C/T | 0.14 | 0.49 | -0.44 | 1.42 | 0.305 |
| 2:211540507 | rs1047891 |  |  |  | C/A | 0.69 | 0.08 | -0.62 | 0.78 | 0.821 |
| 2:227100698 | rs2972146 |  |  |  | G/T | 0.37 | 0.77 | 0.10 | 1.44 | 0.025 |
| 2:57792580 | rs11613352 |  |  |  | T/C | 0.25 | 1.05 | 0.29 | 1.80 | 0.007 |
| 3:11400249 | rs2606736 |  |  |  | C/T | 0.37 | 0.25 | -0.41 | 0.91 | 0.465 |
| 3:119560606 | rs6805251 |  |  |  | T/C | 0.40 | 0.12 | -0.54 | 0.78 | 0.719 |
| 3:132163200 | rs17404153 |  |  |  | G/T | 0.88 | -0.06 | -1.06 | 0.94 | 0.910 |
| 3:47061183 | rs2290547 |  |  |  | G/A | 0.82 | 0.29 | -0.58 | 1.15 | 0.515 |
| 3:50129399 | rs2013208 |  |  |  | T/C | 0.51 | 0.50 | -0.16 | 1.15 | 0.137 |
| 3:52532118 | rs13326165 |  |  |  | A/G | 0.21 | 0.21 | -0.60 | 1.02 | 0.611 |
| 4:100014805 | rs2602836 |  |  |  | A/G | 0.43 | -0.13 | -0.78 | 0.52 | 0.696 |
| 4:103188709 | rs13107325 |  |  |  | C/T | 0.94 | 1.06 | -0.30 | 2.42 | 0.127 |
| 4:26062990 | rs10019888 |  |  |  | A/G | 0.84 | 0.57 | -0.31 | 1.44 | 0.205 |
| 4:89741269 | rs3822072 |  |  |  | G/A | 0.53 | 0.25 | -0.40 | 0.90 | 0.459 |
| 5:53298025 | rs6450176 |  |  |  | G/A | 0.76 | -0.31 | -1.06 | 0.45 | 0.425 |
| 6:127436064 | rs1936800 |  |  |  | C/T | 0.48 | -0.13 | -0.79 | 0.52 | 0.687 |
| 6:139829666 | rs605066 | rs634869 | 1.000 |  | C/T | 0.58 | 0.11 | -0.55 | 0.78 | 0.741 |
| 6:43757896 | rs998584 |  |  |  | C/A | 0.53 | -0.51 | -0.80 | 0.50 | 0.654 |
| 7:130433384 | rs4731702 |  |  |  | T/C | 0.49 | 0.74 | 0.10 | 1.39 | 0.024 |
| 7:150529449 | rs17173637 |  |  |  | T/C | 0.89 | 0.14 | -0.91 | 1.20 | 0.792 |
| 7:17919258 | rs4142995 |  |  |  | G/T | 0.58 | 0.69 | 0.03 | 1.35 | 0.041 |
| 7:50305863 | rs4917014 |  |  |  | G/T | 0.32 | -0.12 | -0.82 | 0.57 | 0.724 |
| 7:6449272 | rs702485 |  |  |  | G/A | 0.43 | 0.45 | -0.20 | 1.11 | 0.177 |
| 7:72982874 | rs17145738 |  |  |  | T/C | 0.12 | 0.93 | -0.09 | 1.94 | 0.075 |
| 8:116599199 | rs2293889 |  |  |  | G/T | 0.58 | 0.45 | -0.21 | 1.11 | 0.184 |
| 8:126490972 | rs2954029 |  |  |  | T/A | 0.49 | 0.30 | -0.35 | 0.94 | 0.371 |
| 8:19844222 | rs12678919 |  |  |  | G/A | 0.09 | 3.44 | 2.29 | 4.58 | 4.1*10^-09^ |
| 8:9183358 | rs9987289 |  |  |  | G/A | 0.92 | 2.11 | 0.94 | 3.28 | 4.0*10^-04^ |
| 9:107664301 | rs1883025 |  |  |  | C/T | 0.76 | 1.22 | 0.46 | 1.97 | 0.002 |
| 9:15305378 | rs581080 |  |  |  | C/G | 0.81 | 0.85 | 0.02 | 1.68 | 0.044 |
| 10:46013277 | rs970548 |  |  |  | C/A | 0.25 | 0.52 | -0.23 | 1.27 | 0.177 |
| 11:10388782 | rs2923084 |  |  |  | A/G | 0.82 | 0.73 | -0.11 | 1.58 | 0.089 |
| 11:116648917 | rs964184 |  |  | 0.998 | C/G | 0.85 | 2.50 | 1.58 | 3.41 | 9.2*10^-08^ |
| 11:122522375 | rs7941030 |  |  |  | C/T | 0.41 | 0.20 | -0.47 | 0.86 | 0.559 |
| 11:46743247 | rs3136441 |  |  |  | C/T | 0.1 | 1.69 | 0.77 | 2.61 | 3.3*10^-04^ |
| 11:51512090 | rs11246602 |  |  |  | C/T | 0.13 | 0.23 | -1.22 | 0.75 | 0.641 |
| 11:61569830 | rs174546 |  |  |  | C/T | 0.68 | 0.26 | -0.43 | 0.95 | 0.458 |
| 11:65391317 | rs12801636 |  |  |  | A/G | 0.22 | 0.13 | -0.66 | 0.91 | 0.750 |
| 11:75455021 | rs499974 |  |  |  | C/A | 0.81 | 1.34 | 0.50 | 2.18 | 0.002 |
| 12:110000193 | rs7134594 | rs2241213 | 0.935 |  | G/A | 0.54 | 0.26 | -0.40 | 0.92 | 0.444 |
| 12:123796238 | rs4759375 | rs10773003 | 1.000 |  | A/G | 0.10 | 0.73 | -0.37 | 1.83 | 0.193 |
| 12:124460167 | rs4765127 | rs7975482 | 1.000 |  | G/A | 0.33 | 0.37 | -0.32 | 1.05 | 0.297 |
| 12:125261593 | rs838880 |  |  |  | C/T | 0.32 | 0.54 | -0.17 | 1.24 | 0.135 |
| 12:20473758 | rs7134375 |  |  |  | A/C | 0.43 | -0.11 | -0.77 | 0.55 | 0.749 |
| 14:105277209 | rs4983559 |  |  |  | G/A | 0.42 | 0.64 | -0.02 | 1.31 | 0.057 |
| 15:58683366 | rs1532085 |  |  |  | A/G | 0.36 | 1.97 | 1.30 | 2.64 | 9.8*10^-09^ |
| 15:63396867 | rs2652834 |  |  |  | G/A | 0.79 | 1.10 | 0.30 | 1.90 | 0.007 |
| 16:53809247 | rs1121980 |  |  |  | G/A | 0.56 | -0.11 | -0.77 | 0.54 | 0.732 |
| 16:56993324 | rs3764261 |  |  |  | A/C | 0.31 | 3.23 | 2.54 | 3.92 | 7.3*10^-20^ |
| 16:67928042 | rs16942887 |  |  |  | A/G | 0.14 | 0.78 | -0.15 | 1.71 | 0.101 |
| 16:81534790 | rs2925979 |  |  |  | C/T | 0.70 | 0.05 | -0.65 | 0.76 | 0.881 |
| 17:37813856 | rs11869286 |  |  |  | C/G | 0.66 | 0.60 | -0.09 | 1.29 | 0.090 |
| 17:66875294 | rs4148008 |  |  |  | C/G | 0.71 | -0.07 | -0.79 | 0.65 | 0.852 |
| 17:76403984 | rs4129767 |  |  |  | A/G | 0.50 | 0.61 | -0.04 | 1.27 | 0.066 |
| 18:47160953 | rs7241918 | rs7240405 | 1.000 |  | G/A | 0.84 | 2.37 | 1.49 | 3.25 | 1.4*10^-07^ |
| 18:57849023 | rs12967135 |  |  |  | G/A | 0.76 | 0.55 | -0.21 | 1.32 | 0.156 |
| 19:11347493 | rs737337 |  |  |  | T/C | 0.91 | 0.56 | -0.60 | 1.72 | 0.348 |
| 19:33899065 | rs731839 |  |  |  | A/G | 0.65 | -0.16 | -0.85 | 0.53 | 0.646 |
| 19:45422946 | rs4420638 |  |  | 0.997 | A/G | 0.83 | 1.10 | 0.24 | 1.97 | 0.013 |
| 19:52324216 | rs17695224 |  |  |  | G/A | 0.73 | 0.01 | -0.71 | 0.74 | 0.968 |
| 19:54792761 | rs386000 |  |  |  | C/G | 0.20 | 0.93 | 0.10 | 1.76 | 0.029 |
| 19:8433196 | rs7255436 | rs2278236 | 0.965 |  | A/G | 0.53 | 0.08 | -0.57 | 0.74 | 0.804 |
| 20:43042364 | rs1800961 |  |  |  | C/T | 0.97 | 2.27 | 0.22 | 4.33 | 0.030 |
| 20:44554015 | rs6065906 |  |  |  | T/C | 0.81 | 1.11 | 0.28 | 1.94 | 0.009 |
| 22:21932068 | rs181362 |  |  |  | C/T | 0.80 | 0.79 | -0.02 | 1.61 | 0.057 |
| **GES_HDL-C-EXT_** | | | |  |  |  |  |  |  |  |
| 1:107549245 | rs2878349 |  |  |  | G/A | 0.69 |  |  |  |  |
| 1:110470764 | rs333947 |  |  |  | G/A | 0.84 |  |  |  |  |
| 1:184049978 | rs10911505 |  |  |  | T/C | 0.69 |  |  |  |  |
| 1:205719532 | rs823114 |  |  |  | A/G | 0.56 |  |  |  |  |
| 1:93837780 | rs145882729 | rs12133576 | 1.000 |  | A/G | 0.36 |  |  |  |  |
| 2:146371961 | rs13396091 |  |  |  | G/A | 0.53 |  |  |  |  |
| 2:188003118 | rs17576323 |  |  |  | C/T | 0.23 |  |  |  |  |
| 2:219699999 | rs78058190 |  |  |  | G/A | 0.94 |  |  |  |  |
| 2:3634753 | rs4850047 |  |  |  | T/C | 0.14 |  |  |  |  |
| 2:66665146 | rs2280334 | rs756292 | 0.824 |  | C/T | 0.68 |  |  |  |  |
| 3:123070426 | rs7614016 | rs11717195 | 0.986 |  | T/C | 0.78 |  |  |  |  |
| 3:152171870 | rs3773910 |  |  |  | G/C | 0.26 |  |  |  |  |
| 3:185803532 | rs112545201 | rs4234589 | 1.000 |  | A/G | 0.87 |  |  |  |  |
| 3:24293001 | rs2044753 | rs9812189 | 0.856 |  | A/G | 0.88 |  |  |  |  |
| 3:36979042 | rs6777217 |  |  |  | G/A | 0.55 |  |  |  |  |
| 4:145659064 | rs11727676 |  |  |  | T/C | 0.91 |  |  |  |  |
| 4:154224048 | rs17369400 |  |  |  | A/G | 0.89 |  |  |  |  |
| 4:858332 | rs11248051 |  |  |  | C/T | 0.90 |  |  |  |  |
| 5:118729286 | rs1045241 |  |  |  | T/C | 0.31 |  |  |  |  |
| 5:158020425 | rs1651274 | rs2914228 | 0.989 |  | C/T | 0.77 |  |  |  |  |
| 5:158022041 | rs2434612 |  |  |  | A/G | 0.80 |  |  |  |  |
| 5:170459675 | rs7730898 |  |  |  | G/A | 0.27 |  |  |  |  |
| 5:72926514 | rs6881956 | rs2544642 | 0.981 |  | A/G | 0.72 |  |  |  |  |
| 6:153459444 | rs1281955 | rs1281988 | 0.829 |  | A/G | 0.39 |  |  |  |  |
| 6:41934514 | rs4130023 |  |  |  | C/T | 0.78 |  |  |  |  |
| 6:42928461 | rs10948059 | rs3805946 | 0.87 |  | G/A | 0.48 |  |  |  |  |
| 7:12269417 | rs3173615 | rs6966915 | 1.000 |  | C/T | 0.60 |  |  |  |  |
| 7:38277792 | rs2534596 |  |  |  | G/A | 0.38 |  |  |  |  |
| 8:121868551 | rs4871137 |  |  |  | G/T | 0.34 |  |  |  |  |
| 8:144303418 | rs10087900 |  |  |  | G/A | 0.57 |  |  |  |  |
| 8:29360305 | rs10091038 |  |  |  | A/C | 0.43 |  |  |  |  |
| 8:6599005 | rs2936512 |  |  |  | C/T | 0.66 |  |  |  |  |
| 8:71260460 | rs10504474 |  |  |  | G/A | 0.90 |  |  |  |  |
| 8:72395582 | rs200441621 | rs7014001 | 0.894 |  | T/C | 0.94 |  |  |  |  |
| 10:122859177 | rs7079858 | rs2918605 | 0.938 |  | A/G | 0.90 |  |  |  |  |
| 10:122900623 | rs2901286 | rs12219793 | 0.932 |  | G/T | 0.01 |  |  |  |  |
| 10:8576206 | rs4749779 |  |  |  | G/A | 0.30 |  |  |  |  |
| 11:109979945 | rs10789752 |  |  |  | T/C | 0.30 |  |  |  |  |
| 11:13355770 | rs6486121 |  |  |  | C/T | 0.39 |  |  |  |  |
| 11:27675712 | rs1519480 |  |  |  | T/C | 0.71 |  |  |  |  |
| 11:45913607 | rs77403571 |  |  | 1.000 | A/G | 0.06 |  |  |  |  |
| 12:101873956 | rs2373459 |  |  |  | T/C | 0.68 |  |  |  |  |
| 12:26474867 | rs10842708 | rs7132434 | 1.000 |  | G/A | 0.74 |  |  |  |  |
| 12:33459554 | rs4551851 | rs1905423 | 0.996 |  | A/C | 0.44 |  |  |  |  |
| 14:92498829 | rs58752567 | rs12588287 | 1.000 |  | C/G | 0.25 |  |  |  |  |
| 16:11454650 | rs7188861 |  |  |  | A/C | 0.21 |  |  |  |  |
| 17:486821 | rs12938449 | rs12951098 | 0.823 |  | A/G | 0.82 |  |  |  |  |
| 18:21161134 | rs1788783 |  |  |  | T/C | 0.48 |  |  |  |  |
| 19:3414088 | rs12975319 |  |  |  | G/A | 0.67 |  |  |  |  |
| 20:30184866 | rs6120815 |  |  |  | C/T | 0.17 |  |  |  |  |
| 20:51263786 | rs856404 |  |  |  | G/A | 0.31 |  |  |  |  |
| 21:36343552 | rs2834707 | rs9974986 | 0.858 |  | C/T | 0.66 |  |  |  |  |

^1^Position of build 37, ^2^Proxy of original SNP, used when original SNP was not genotyped within study population, ^3^Linkage disequilibrium between original and proxy SNP according to 1000 Genome, Phase 3

**Table S3:** Overview of 58 loci associated with LDL-C included into genetic effect allele sum score GES_LDL-C_ based on [14] and 37 loci added to the 58 loci in the genetic effect allele sum score GES_LDL-C-EXT_ based on [15-17].

| **Chr:Position^1^** | **Original SNP** | **Proxy SNP^2^** | **Linkage disequi-librium^3^ (LD=r^2^)** | **Imputation quality** | **Effect allele/ Non-effect allele** | **Effect allele frequency** | **Effect of effect allele** | **Lower 95% CI** | **Upper 95% CI** | **P-value** |
| --- | --- | --- | --- | --- | --- | --- | --- | --- | --- | --- |
| **GES_LDL-C_** | | | |  |  |  |  |  |  |  |
| 1:109818306 | rs629301 |  |  |  | T/G | 0.77 | 5.20 | 3.42 | 6.99 | 1.2*10^-08^ |
| 1:150958836 | rs267733 |  |  |  | A/G | 0.85 | 1.45 | -0.61 | 3.52 | 0.168 |
| 1:220973563 | rs2642442 |  |  |  | T/C | 0.69 | 1.32 | -0.25 | 2.93 | 0.098 |
| 1:234858597 | rs514230 | rs558971 | 0.967 |  | G/A | 0.53 | 1.61 | 0.15 | 3.07 | 0.031 |
| 1:25775733 | rs12027135 |  |  |  | T/A | 0.56 | 0.81 | -0.68 | 2.29 | 0.287 |
| 1:27138393 | rs12748152 |  |  |  | T/C | 0.08 | 1.11 | -1.66 | 3.87 | 0.432 |
| 1:55504650 | rs2479409 |  |  |  | G/A | 0.35 | 1.28 | -0.28 | 2.85 | 0.109 |
| 1:63025942 | rs2131925 |  |  |  | T/G | 0.68 | 1.11 | -0.51 | 2.72 | 0.179 |
| 2:118835841 | rs10490626 |  |  |  | G/A | 0.93 | 1.70 | -1.21 | 4.61 | 0.251 |
| 2:121309488 | rs2030746 |  |  |  | T/C | 0.41 | -0.05 | -1.58 | 1.49 | 0.950 |
| 2:21263900 | rs1367117 |  |  |  | A/G | 0.32 | 4.62 | 3.00 | 6.24 | 2.3*10^-08^ |
| 2:216304384 | rs1250229 |  |  |  | C/T | 0.73 | 1.61 | -0.06 | 3.29 | 0.059 |
| 2:234679384 | rs11563251 |  |  |  | T/C | 0.09 | 0.47 | -2.13 | 3.06 | 0.724 |
| 2:44072576 | rs4299376 |  |  |  | G/T | 0.31 | 1.76 | 0.14 | 3.38 | 0.034 |
| 2:63149557 | rs2710642 |  |  |  | A/G | 0.66 | 0.80 | -0.78 | 2.39 | 0.320 |
| 3:132163200 | rs17404153 |  |  |  | G/T | 0.88 | -0.39 | -2.69 | 1.91 | 0.738 |
| 3:32533010 | rs7640978 |  |  |  | C/T | 0.93 | -2.71 | -5.54 | 0.12 | 0.061 |
| 4:3473139 | rs6831256 |  |  |  | G/A | 0.42 | 0.04 | -1.45 | 1.54 | 0.954 |
| 5:122855416 | rs4530754 |  |  |  | A/G | 0.56 | 1.11 | -0.40 | 2.63 | 0.149 |
| 5:156390297 | rs6882076 |  |  |  | C/T | 0.62 | 1.38 | -0.15 | 2.91 | 0.078 |
| 5:74656539 | rs12916 |  |  |  | C/T | 0.42 | 1.75 | 0.22 | 3.27 | 0.025 |
| 6:116312893 | rs9488822 | rs3798236 | 1.000 |  | C/T | 0.31 | -0.57 | -2.18 | 1.04 | 0.488 |
| 6:160578860 | rs1564348 |  |  |  | C/T | 0.17 | 3.86 | 1.84 | 5.88 | 1.8*10^-04^ |
| 6:16127407 | rs3757354 |  |  |  | C/T | 0.78 | 1.05 | -0.74 | 2.85 | 0.249 |
| 6:26093141 | rs1800562 |  |  |  | G/A | 0.95 | 1.66 | -1.89 | 5.21 | 0.360 |
| 6:32412435 | rs3177928 |  |  |  | A/G | 0.15 | 2.40 | 0.28 | 4.53 | 0.027 |
| 7:21607352 | rs12670798 |  |  |  | C/T | 0.24 | 1.62 | -0,15 | 3.38 | 0.072 |
| 7:25991826 | rs4722551 |  |  |  | C/T | 0.16 | 1.89 | -0.14 | 3.92 | 0.068 |
| 7:44579180 | rs2072183 |  |  |  | C/G | 0.23 | 1.37 | -0.41 | 3.14 | 0.132 |
| 8:126490972 | rs2954029 |  |  |  | A/T | 0.51 | 1.81 | 0.33 | 3.30 | 0.017 |
| 8:145043543 | rs11136341 | rs7832643 | 0.805 |  | T/G | 0.38 | 0.58 | -0.95 | 2.11 | 0.459 |
| 8:55421614 | rs10102164 |  |  |  | A/G | 0.19 | 0.94 | -0.95 | 2.84 | 0.329 |
| 8:59388565 | rs2081687 | rs13277801 | 0.845 |  | C/T | 0.36 | 0.38 | -1.18 | 1.95 | 0.632 |
| 8:9183358 | rs9987289 |  |  |  | G/A | 0.92 | 1.59 | -1.09 | 4.28 | 0.245 |
| 9:136155000 | rs9411489 |  |  |  | T/C | 0.22 | 2.70 | 0.89 | 4.51 | 0.003 |
| 9:2640759 | rs3780181 |  |  |  | A/G | 0.93 | 4.34 | 1.44 | 7.23 | 0.003 |
| 10:11393388 | rs2255141 |  |  |  | A/G | 0.28 | 1.03 | -0.62 | 2.68 | 0.222 |
| 11:116648917 | rs964184 |  |  | 0.998 | G/C | 0.15 | 2.54 | 0.42 | 4.65 | 0.019 |
| 11:126243952 | rs11220462 |  |  |  | A/G | 0.14 | 2.27 | 0.10 | 4.43 | 0.040 |
| 11:61569830 | rs174546 |  |  |  | C/T | 0.68 | 1.55 | -0.04 | 3.13 | 0.056 |
| 12:112072424 | rs11065987 |  |  |  | A/G | 0.54 | 0.18 | -1.33 | 1.70 | 0.814 |
| 12:121416650 | rs1169288 |  |  |  | C/A | 0.34 | 0.80 | -0.77 | 2.37 | 0.321 |
| 13:32953388 | rs4942486 |  |  |  | T/C | 0.47 | -0.13 | -1.62 | 1.36 | 0.864 |
| 14:24883887 | rs8017377 |  |  |  | A/G | 0.47 | 0.43 | -1.08 | 1.93 | 0.578 |
| 16:56993324 | rs3764261 |  |  |  | C/A | 0.69 | 0.23 | -1.37 | 1.83 | 0.776 |
| 16:72108093 | rs2000999 |  |  |  | A/G | 0.21 | 1.04 | -0.79 | 2.87 | 0.268 |
| 17:45425115 | rs7206971 | rs6504872 | 0.935 |  | T/C | 0.48 | 1.88 | 0.40 | 3.36 | 0.013 |
| 17:64210580 | rs1801689 |  |  |  | C/A | 0.03 | 1.22 | -3.08 | 5.52 | 0.579 |
| 17:7091650 | rs314253 |  |  |  | T/C | 0.64 | 1.91 | 0.35 | 3.48 | 0.017 |
| 19:11202306 | rs6511720 |  |  |  | G/T | 0.89 | 6.48 | 4.09 | 8.87 | 1.2*10^-07^ |
| 19:19407718 | rs10401969 |  |  |  | T/C | 0.92 | 2.80 | -0.02 | 5.62 | 0.051 |
| 19:45422946 | rs4420638 |  |  | 0.997 | G/A | 0.17 | 7.32 | 5.34 | 9.31 | 5.3*10^-13^ |
| 20:12962718 | rs364585 |  |  |  | G/A | 0.62 | -0.18 | -1.72 | 1.37 | 0.824 |
| 20:17845921 | rs2328223 |  |  |  | C/A | 0.19 | 1.08 | -0.82 | 2.98 | 0.267 |
| 20:39091487 | rs2902940 |  |  |  | A/G | 0.72 | 0.60 | -1.07 | 2.26 | 0.482 |
| 20:39672618 | rs6029526 | rs6065311 | 1.000 |  | C/T | 0.49 | 1.31 | -0.17 | 2.81 | 0.083 |
| 22:30378703 | rs5763662 |  |  |  | T/C | 0.02 | 1.20 | -3.80 | 6.20 | 0.639 |
| 22:46627603 | rs4253772 |  |  |  | T/C | 0.11 | 0.67 | -1.73 | 3.06 | 0.585 |
| **GES_LDL-C-EXT_** | | | |  |  |  |  |  |  |  |
| 1:10556447 | rs648324 | rs585870 | 0.995 |  | A/C | 0.70 |  |  |  |  |
| 1:107617707 | rs1730859 |  |  |  |  |  |  |  |  |  |
| 1:16509671 | rs7538216 | rs4661718 | 0.988 |  | T/C | 0.64 |  |  |  |  |
| 1:18808292 | rs2992753 | rs2992764 | 0.971 |  | G/A | 0.36 |  |  |  |  |
| 2:109232388 | rs826682 |  |  |  | C/A | 0.14 |  |  |  |  |
| 2:46166321 | rs12712955 |  |  |  | A/G | 0.52 |  |  |  |  |
| 3:122176238 | rs72285796 | rs3762637 | 0.992 |  | T/C | 0.14 |  |  |  |  |
| 3:160086055 | rs10513551 |  |  |  | G/T | 0.45 |  |  |  |  |
| 5:131804045 | rs2522061 | rs2522062 | 0.994 |  | A/G | 0.79 |  |  |  |  |
| 5:141913503 | rs249756 | rs249765 | 0.988 |  | C/A | 0.23 |  |  |  |  |
| 5:52193237 | rs870992 |  |  |  | G/A | 0.10 |  |  |  |  |
| 5:72014569 | rs3010276 |  |  |  | G/A | 0.79 |  |  |  |  |
| 6:100600097 | rs17789218 |  |  |  | T/C | 0.77 |  |  |  |  |
| 6:52452585 | rs2239620 | rs2239619 | 0.923 |  | A/C | 0.63 |  |  |  |  |
| 7:100285974 | rs221797 |  |  |  | A/C | 0.11 |  |  |  |  |
| 7:107140239 | rs2712199 | rs4727681 | 0.902 |  | C/T | 0.30 |  |  |  |  |
| 7:2296552 | rs144787122 |  |  |  | G/A | 0.00 |  |  |  |  |
| 7:87076587 | rs1014283 |  |  |  | C/A | 0.83 |  |  |  |  |
| 8:21937667 | rs6557781 |  |  |  | C/T | 0.86 |  |  |  |  |
| 10:74662593 | rs57176252 | rs16930150 | 1.000 |  | A/G | 0.95 |  |  |  |  |
| 12:109137726 | rs7968419 | rs3741782 | 0.907 |  | G/A | 032 |  |  |  |  |
| 12:40606663 | rs1907631 | rs2723260 | 1.000 |  | T/G | 0.11 |  |  |  |  |
| 12:51055708 | rs1521516 |  |  |  | C/T | 0.64 |  |  |  |  |
| 13:111025118 | rs4773173 | rs9521732 | 0.802 |  | C/A | 0.61 |  |  |  |  |
| 14:70817141 | rs11620731 | rs7150558 | 0.958 |  | T/C | 0.86 |  |  |  |  |
| 15:75289722 | rs3812945 |  |  |  | C/T | 0.46 |  |  |  |  |
| 17:26694861 | rs704 |  |  |  | T/C | 0.47 |  |  |  |  |
| 17:57875554 | rs2645492 | rs7215180 | 0.919 |  | A/G | 0.78 |  |  |  |  |
| 17:73778609 | rs73352129 | rs2125345 | 0.995 |  | T/C | 0.71 |  |  |  |  |
| 18:19907770 | rs79588679 |  |  |  | C/T | 0.84 |  |  |  |  |
| 18:9522606 | rs329007 |  |  |  | A/G | 0.77 |  |  |  |  |
| 19:2814181 | rs941408 |  |  |  | T/C | 0.32 |  |  |  |  |
| 19:4493708 | rs892161 |  |  |  | A/G | 0.39 |  |  |  |  |
| 19:58681861 | rs117492019 |  |  |  | G/T | 0.81 |  |  |  |  |
| 21:33059831 | rs17660708 |  |  |  | C/T | 0.05 |  |  |  |  |
| 22:37462936 | rs855791 |  |  |  | A/G | 0.45 |  |  |  |  |
| 22:50878927 | rs12171249 |  |  |  | G/A | 0.71 |  |  |  |  |

^1^Position of build 37, ^2^Proxy of original SNP, used when original SNP was not genotyped within study population, ^3^Linkage disequilibrium between original and proxy SNP according to 1000 Genome, Phase 3

**Table S4:** Overview of 74 loci associated with TC included into genetic effect allele sum score GES_TC_ based on [14] and 43 loci added to the 74 loci in the genetic effect allele sum score GES_TC-EXT_ based on [15-17].

| **Chr:Position^1^** | **Original SNP** | **Proxy SNP^2^** | **Linkage disequi-librium^3^ (LD=r^2^)** | **Imputation quality** | **Effect allele/ Non-effect allele** | **Effect allele frequency** | **Effect of effect allele** | **Lower 95% CI** | **Upper 95% CI** | **p-value** |
| --- | --- | --- | --- | --- | --- | --- | --- | --- | --- | --- |
| **GES_TC_** | | | |  |  |  |  |  |  |  |
| 1:109818306 | rs629301 |  |  |  | T/G | 0.77 | 4.11 | 2.19 | 6.03 | 2.7*10-05 |
| 1:220973563 | rs2642442 |  |  |  | T/C | 0.69 | 1.17 | -0.53 | 2.88 | 0.178 |
| 1:234858597 | rs514230 | rs558971 | 0.967 |  | G/A | 0.53 | 1.57 | 0.01 | 3.14 | 0.049 |
| 1:23766233 | rs1077514 |  |  |  | T/C | 0.86 | -2.18 | -4.45 | 0.10 | 0.061 |
| 1:2577573 | rs12027135 |  |  |  | T/A | 0.56 | 1.49 | -0.10 | 3.08 | 0.067 |
| 1:55504650 | rs2479409 |  |  |  | G/A | 0.35 | 1.41 | -0.28 | 3.09 | 0.101 |
| 1:63025942 | rs2131925 |  |  |  | T/G | 0.68 | 2.44 | 0.71 | 4.17 | 0.006 |
| 1:93009438 | rs7515577 | rs6690764 | 1.000 |  | A/G | 0.80 | 0.30 | -1.70 | 2.30 | 0.770 |
| 2:118835841 | rs10490626 |  |  |  | G/A | 0.93 | 2.18 | -0.94 | 5.31 | 0.171 |
| 2:121309488 | rs2030746 |  |  |  | T/C | 0.41 | -0.09 | -1.73 | 1.56 | 0.918 |
| 2:135837906 | rs7570971 |  |  |  | A/C | 0.44 | 0.75 | -0.86 | 2.36 | 0.362 |
| 2:169830155 | rs2287623 |  |  |  | G/A | 0.40 | 0.53 | -1.12 | 2.18 | 0.529 |
| 2:203532304 | rs11694172 |  |  |  | G/A | 0.24 | -0.18 | -2.04 | 1.68 | 0.849 |
| 2:21263900 | rs1367117 |  |  |  | A/G | 0.32 | 4.82 | 3.09 | 6.56 | 5.5*10^-08^ |
| 2:234679384 | rs11563251 |  |  |  | T/C | 0.09 | 2.02 | -0.77 | 4.80 | 0.156 |
| 2:27730940 | rs1260326 |  |  |  | T/C | 0.42 | 1.46 | -0.16 | 3.07 | 0.077 |
| 2:44072576 | rs4299376 |  |  |  | G/T | 0.31 | 2.29 | 0.55 | 4.03 | 0.010 |
| 3:12628920 | rs2290159 | rs11709504 | 0.800 |  | T/C | 0.82 | 0.39 | -1.69 | 2.48 | 0.710 |
| 3:32533010 | rs7640978 |  |  |  | C/T | 0.93 | -2.61 | -5.65 | 0.43 | 0.093 |
| 3:58381287 | rs13315871 |  |  |  | G/A | 0.90 | 1.05 | -1.64 | 3.74 | 0.444 |
| 4:3473139 | rs6831256 |  |  |  | G/A | 0.42 | 0.70 | -0.91 | 2.31 | 0.394 |
| 5:122855416 | rs4530754 |  |  |  | A/G | 0.56 | 1.48 | -0.14 | 3.11 | 0.073 |
| 5:156390297 | rs6882076 |  |  |  | C/T | 0.62 | 1.14 | -0.50 | 2.79 | 0.173 |
| 5:74656539 | rs12916 |  |  |  | C/T | 0.42 | 2.00 | 0.37 | 3.64 | 0.016 |
| 6:116312893 | rs9488822 | rs3798236 | 1.000 |  | C/T | 0.31 | -1.43 | -3.16 | 0.30 | 0.106 |
| 6:135411228 | rs9376090 |  |  |  | T/C | 0.73 | 2.70 | 0.89 | 4.50 | 0.003 |
| 6:160578860 | rs1564348 |  |  |  | C/T | 0.17 | 4.84 | 2.67 | 7.00 | 1.3*10^-05^ |
| 6:16127407 | rs3757354 |  |  |  | C/T | 0.78 | 1.12 | -0.80 | 3.05 | 0.252 |
| 6:26093141 | rs1800562 |  |  |  | G/A | 0.95 | 1.58 | -2.23 | 5.39 | 0.417 |
| 6:32412435 | rs3177928 |  |  |  | A/G | 0.15 | 2.88 | 0.60 | 5.16 | 0.013 |
| 6:34546560 | rs2814982 |  |  |  | C/T | 0.88 | 0.16 | -1.86 | 3.07 | 0.629 |
| 6:3925083 | rs2758886 |  |  |  | A/G | 0.28 | -0.58 | -2.34 | 1.19 | 0.521 |
| 7:1083777 | rs1997243 |  |  |  | G/A | 0.18 | 0.76 | -1.35 | 2.87 | 0.481 |
| 7:21607352 | rs12670798 |  |  |  | C/T | 0.24 | 2.29 | 0.40 | 4.18 | 0.018 |
| 7:25991826 | rs4722551 |  |  |  | C/T | 0.16 | 1.57 | -0.61 | 3.75 | 0.159 |
| 7:44579180 | rs2072183 |  |  |  | C/G | 0.23 | 1.77 | -0.13 | 3.68 | 0.069 |
| 8:126490972 | rs2954029 |  |  |  | A/T | 0.51 | 2.12 | 0.52 | 3.71 | 0.009 |
| 8:145043543 | rs11136341 | rs7832643 | 0.805 |  | T/G | 0.38 | 0.86 | -0.79 | 2.51 | 0.307 |
| 8:18272881 | rs1495741 | rs4921914 | 1.000 |  | C/T | 0.23 | -0.45 | -2.36 | 1.45 | 0.640 |
| 8:55421614 | rs10102164 |  |  |  | A/G | 0.19 | 0.19 | -1.84 | 2.23 | 0.852 |
| 8:59388565 | rs2081687 | rs13277801 | 0.845 |  | C/T | 0.36 | 0.32 | -1.36 | 2.01 | 0.706 |
| 8:9183358 | rs9987289 |  |  |  | G/A | 0.92 | 3.67 | 0.79 | 6.54 | 0.012 |
| 9:107664301 | rs1883025 |  |  |  | C/T | 0.76 | 2.30 | 0.43 | 4.16 | 0.016 |
| 9:136155000 | rs9411489 |  |  |  | T/C | 0.22 | 2.31 | 0.37 | 4.26 | 0.020 |
| 9:15305378 | rs581080 |  |  |  | C/G | 0.81 | 0.76 | -1.28 | 2.79 | 0.466 |
| 9:2640759 | rs3780181 |  |  |  | A/G | 0.93 | 4.31 | 1.21 | 7.41 | 0.006 |
| 10:113933886 | rs2255141 |  |  |  | A/G | 0.28 | 1.16 | -0.61 | 2.93 | 0.201 |
| 10:17260290 | rs10904908 |  |  |  | G/A | 0.42 | 1.20 | -0.39 | 2.80 | 0.140 |
| 10:46013277 | rs970548 |  |  |  | C/A | 0.25 | 1.45 | -0.39 | 3.30 | 0.123 |
| 11:116648917 | rs964184 |  |  | 0.998 | G/C | 0.15 | 2.54 | 0.42 | 4.65 | 0.019 |
| 11:118486067 | rs11603023 |  |  |  | T/C | 0.41 | -0.48 | -2.13 | 1.16 | 0.564 |
| 11:122522375 | rs7941030 |  |  |  | C/T | 0.41 | 1.38 | -0.26 | 3.01 | 0.099 |
| 11:126243952 | rs11220462 |  |  |  | A/G | 0.14 | 1.60 | -0.73 | 3.92 | 0.178 |
| 11:18632984 | rs10128711 |  |  |  | C/T | 0.74 | 1.57 | -0.25 | 3.40 | 0.091 |
| 11:61569830 | rs174546 |  |  |  | C/T | 0.68 | 2.29 | 0.59 | 3.99 | 0.008 |
| 12:112072424 | rs11065987 |  |  |  | A/G | 0.54 | 0.91 | -0.72 | 2.53 | 0.274 |
| 12:121416650 | rs1169288 |  |  |  | C/A | 0.34 | 1.51 | -0.17 | 3.20 | 0.079 |
| 12:9082581 | rs4883201 |  |  |  | A/G | 0.89 | 0.69 | -1.86 | 3.23 | 0.597 |
| 15:58683366 | rs1532085 |  |  |  | A/G | 0.36 | 1.09 | -0.57 | 2.74 | 0.200 |
| 16:56993324 | rs3764261 |  |  |  | A/C | 0.31 | 1.86 | 0.15 | 3.58 | 0.033 |
| 16:72108093 | rs2000999 |  |  |  | A/G | 0.21 | 0.61 | -1.35 | 2.58 | 0.541 |
| 17:45425115 | rs7206971 | rs6504872 | 0.935 |  | T/C | 0.48 | 1.58 | -0.02 | 3.17 | 0.053 |
| 17:7091650 | rs314253 |  |  |  | T/C | 0.64 | 2.64 | 0.96 | 4.31 | 0.002 |
| 18:47160953 | rs7241918 | rs7240405 | 1.000 |  | G/A | 0.84 | 2.31 | 0.13 | 4.48 | 0.038 |
| 19:11202306 | rs6511720 |  |  |  | G/T | 0.89 | 6.22 | 3.65 | 8.79 | 2.2*10^-06^ |
| 19:19407718 | rs10401969 |  |  |  | T/C | 0.92 | 4.39 | 1.37 | 7.41 | 0.004 |
| 19:45422946 | rs4420638 |  |  | 0.997 | G/A | 0.17 | 7.32 | 5.34 | 9.31 | 5.3*10^-13^ |
| 19:49206417 | rs492602 |  |  |  | G/A | 0.41 | -0.20 | -1.81 | 1.42 | 0.810 |
| 20:34152782 | rs2277862 |  |  |  | C/T | 0.85 | 1.16 | -1.08 | 3.39 | 0.311 |
| 20:39091487 | rs2902940 |  |  |  | A/G | 0.72 | 0.94 | -0.85 | 2.73 | 0.301 |
| 20:39672618 | rs6029526 | rs6065311 | 1.000 |  | C/T | 0.49 | 2.06 | 0.46 | 3.66 | 0.012 |
| 20:43042364 | rs1800961 |  |  |  | C/T | 0.97 | 6.71 | 1.65 | 11.78 | 0.009 |
| 22:35711098 | rs138777 |  |  |  | A/G | 0.35 | 1.05 | -0.62 | 2.73 | 0.217 |
| 22:46627603 | rs4253772 |  |  |  | T/C | 0.11 | 0.32 | -2.25 | 2.89 | 0.808 |
| **GES_TC-EXT_** | | | |  |  |  |  |  |  |  |
| 1:10556447 | rs648324 | rs585870 | 0.995 |  | A/C | 0.70 |  |  |  |  |
| 1:113190807 | rs17030613 |  |  |  | A/C | 0.80 |  |  |  |  |
| 1:120257576 | rs477992 |  |  |  | G/A | 0.69 |  |  |  |  |
| 1:16515805 | rs6656611 |  |  |  | C/T | 0.62 |  |  |  |  |
| 1:182970547 | rs4651135 | rs4454510 | 1.000 |  | A/G | 0.53 |  |  |  |  |
| 1:236718620 | rs1126627 | rs16834016 | 0.852 |  | C/A | 0.30 |  |  |  |  |
| 1:28298951 | rs6699701 |  |  |  | C/T | 0.71 |  |  |  |  |
| 2:111600519 | rs13395354 |  |  |  | T/C | 0.18 |  |  |  |  |
| 2:113867288 | rs55709272 | rs6734238 | 0.873 |  | A/G | 0.60 |  |  |  |  |
| 2:163110536 | rs2111485 |  |  |  | A/G | 0.38 |  |  |  |  |
| 2:17930195 | rs6761104 | rs7574887 | 0.950 |  | T/C | 0.89 |  |  |  |  |
| 2:46166321 | rs12712955 |  |  |  | A/G | 0.52 |  |  |  |  |
| 3:122176238 | rs72285796 | rs3762637 | 0.992 |  | C/T | 0.86 |  |  |  |  |
| 3:142625778 | rs1470121 | rs55940906 | 0.996 |  | C/T | 0.64 |  |  |  |  |
| 3:160171092 | rs56394279 | rs2279457 | 0.965 |  | G/A | 0.55 |  |  |  |  |
| 3:64706499 | rs9870322 | rs9864077 | 0.935 |  | T/C | 0.70 |  |  |  |  |
| 4:40428010 | rs278981 |  |  |  | G/A | 0.76 |  |  |  |  |
| 5:131804045 | rs2522061 | rs2522062 | 0.994 |  | A/G | 0.79 |  |  |  |  |
| 5:52193237 | rs870992 |  |  |  | G/A | 0.10 |  |  |  |  |
| 5:72014569 | rs3010276 |  |  |  | G/A | 0.79 |  |  |  |  |
| 6:151858598 | rs9371220 | rs13213582 | 1.000 |  | T/C | 0.10 |  |  |  |  |
| 6:20405539 | rs6456350 | rs9368188 | 0.932 |  | G/A | 0.66 |  |  |  |  |
| 6:37038432 | rs913499 |  |  |  | A/G | 0.47 |  |  |  |  |
| 6:52453220 | rs2239619 |  |  |  | A/C | 0.63 |  |  |  |  |
| 6:53497222 | rs1326543 |  |  |  | G/A | 0.26 |  |  |  |  |
| 7:100422481 | rs314311 |  |  |  | C/A | 0.22 |  |  |  |  |
| 7:87076587 | rs1014283 |  |  |  | C/A | 0.83 |  |  |  |  |
| 7:92408370 | rs445 |  |  |  | T/C | 0.10 |  |  |  |  |
| 8:21937667 | rs6557781 |  |  |  | C/T | 0.86 |  |  |  |  |
| 8:74881674 | rs7013120 |  |  |  | G/A | 0.72 |  |  |  |  |
| 9:22088094 | rs10738607 | rs10757274 | 0.969 |  | A/G | 0.54 |  |  |  |  |
| 10:102027407 | rs12784396 | rs2862954 | 0.805 |  | C/T | 0.48 |  |  |  |  |
| 11:77973980 | rs2511158 | rs1017908 | 0.993 |  | T/C | 0.84 |  |  |  |  |
| 12:100850750 | rs7955221 | rs10860595 | 0.880 |  | A/G | 0.58 |  |  |  |  |
| 12:25408464 | rs12320328 |  |  |  | A/G | 0.92 |  |  |  |  |
| 13:111025118 | rs4773173 |  |  | 1.000 | A/G | 0.66 |  |  |  |  |
| 13:41609047 | rs17532301 |  |  |  | G/A | 0.94 |  |  |  |  |
| 14:75278211 | rs12588415 |  |  |  | G/A | 0.54 |  |  |  |  |
| 17:28574177 | rs7214248 | rs1050565 | 0.969 |  | A/G | 0.67 |  |  |  |  |
| 17:29699416 | rs2854322 |  |  |  | T/C | 0.69 |  |  |  |  |
| 18:9526184 | rs328996 | rs329007 | 1.000 |  | A/G | 0.77 |  |  |  |  |
| 19:2814181 | rs941408 |  |  |  | T/C | 0.32 |  |  |  |  |
| 20:31392777 | rs6058893 |  |  |  | T/C | 0.39 |  |  |  |  |

^1^Position of build 37, ^2^Proxy of original SNP, used when original SNP was not genotyped within study population, ^3^Linkage disequilibrium between original and proxy SNP according to 1000 Genome, Phase 3

**Table S5:** Pearson correlation coefficient of high-density lipoprotein cholesterol (HDL-C), low-density lipoprotein cholesterol (LDL-C), total cholesterol (TC), GES_HDL-C_, GES_LDL-C_, GES_TC_ based on [14], education (years of training) and income (€/month).

|  | **HDL-C** | **LDL-C** | **TC** | **GES_HDL-C_** | **GES_LDL-C_** | **GES_TC_** | **Education** | **Income** |
| --- | --- | --- | --- | --- | --- | --- | --- | --- |
| **HDL-C** | 1 | -0.08 | 0.19 | 0.17 | -0.02 | 0.06 | -0.06 | -0.01 |
| **LDL-C** | -0.08 | 1 | 0.84 | -0.05 | 0.17 | 0.16 | -0.05 | -0.05 |
| **TC** | 0.19 | 0.84 | 1 | 0.01 | 0.17 | 0.19 | -0.07 | -0.04 |
| **GES_HDL-C_** | 0.17 | -0.05 | 0.01 | 1 | -0.06 | 0.07 | -0.01 | -0.01 |
| **GES_LDL-C_** | 0.17 | 0.17 | 0.17 | -0.06 | 1 | 0.70 | 0.02 | -0.02 |
| **GES_TC_** | 0.06 | 0.16 | 0.19 | 0.07 | 0.70 | 1 | 0.00 | -0.03 |
| **Education** | -0.06 | -0.05 | -0.07 | -0.01 | 0.02 | 0.00 | 1 | 0.45 |
| **Income** | -0.01 | -0.05 | -0.04 | -0.01 | -0.02 | -0.03 | 0.45 | 1 |

**Table S6**: Sex- and age- adjusted effects per GES_Lipid-EXT_ standard deviation and corresponding 95% confidence intervals (95% CI) on high-density lipoprotein cholesterol (HDL-C), low-density lipoprotein cholesterol (LDL-C) and total cholesterol (TC) in linear regression models including main effects of a lipid-associated genetic effect allele sum score (GES_Lipid-EXT_) based on [14-17].

|  | HDL-C | | | LDL-C | | | TC | | |
| --- | --- | --- | --- | --- | --- | --- | --- | --- | --- |
| Lipid ~ GES_Lipid-EXT_ + age + sex | | | | | | | | | |
| Intercept | 4515 | -27.66 (-37.74; -17.59) | 7.7*10^-08^ | 4502 | 35.30 (16.53; 54.07) | 2.3*10^-04^ | 4516 | 62.38 (39.31; 85.46) | 1.2*10^-07^ |
| Age |  | 0.02 (-0.04; 0.08) | 0.444 |  | 0.28 (0.15; 0.42) | 3.0*10^-05^ |  | 0.35 (0.21; 0.49) | 1.5*10^-06^ |
| Sex |  | 14.08 (13.17; 14.98) | 2.4*10^-186^ |  | 0.20 (-1.88; 2.29) | 0.847 |  | 7.81 (5.58; 10.05) | 7.6*10^-12^ |
| GES_Lipid-EXT_ |  | 3.04 (2.64; 3.50) | 1.6*10^-39^ |  | 5.84 (4.78; 6.90) | 1.3*10^-27^ |  | 7.00 (5.87; 8.12) | 2.1*10^-34^ |

**Table S7**: Sex- and age- adjusted effects per GES_Lipid-EXT_ standard deviation and corresponding 95% confidence intervals (95% CI) on high-density lipoprotein cholesterol (HDL-C), low-density lipoprotein cholesterol (LDL-C) and total cholesterol (TC) in linear regression models including main effects of a lipid-associated genetic effect allele sum score (GES_Lipid-EXT_) based on [14-17], indicators of socioeconomic position (SEP; education groups and income tertiles) and interaction terms of GES_Lipid-EXT_ and indicators of SEP

|  | HDL-C | | | LDL-C | | | TC | | |
| --- | --- | --- | --- | --- | --- | --- | --- | --- | --- |
| Lipid ~ age + sex + GES_Lipid-EXT_ + Education + GES_Lipid-EXT_*Education | | | | | | | | | |
|  | **n** | **β (95%-CI)** | ***p*** | **n** | **β (95%-CI)** | ***p*** | **n** | **β (95%-CI)** | ***p*** |
| Intercept | 4502 | -26.90 (-43.46; -10.34) | 1.5*10^-03^ | 4489 | 31.57 (0.26; 62.88) | 0.048 | 4503 | 54.71 (15.85; 93.57) | 5.8*10^-03^ |
| Age |  | 0.05 (-0.01; 0.11) | 0.072 |  | 0.26 (0.12; 0.40) | 2.0*10^-04^ |  | 0.33 (0.19; 0.48) | 7.3*10^-06^ |
| Sex |  | 14.82 (13.87; 15.78) | 1.1*10^-184^ |  | -0.86 (-3.08; 1.35) | 0.444 |  | 7.17 (4.80; 9.54) | 3,3*10^-09^ |
| GES_Lipid-EXT_ |  | 2.97 (2.18; 3.76) | 1.6*10^.13^ |  | 6.14 (4.25; 8.02) | 2.0*10^-10^ |  | 7.46 (5.48; 9.44) | 1.4*10^-13^ |
| Education (low) |  | -1.90 (-33.59; 29.80) | 0.907 |  | 42.88 (-14.51; 100.28) | 0.143 |  | 42.49 (-28.49; 113.46) | 0.241 |
| Education |  | -6.83 (-27.39; 13.72) | 0.515 |  | 3.23 (-33.98; 40.45) | 0.865 |  | 8.60 (-38.91; 56.10) | 0.723 |
| Education (high) |  | ref. | - |  | ref. | - |  | ref. | - |
| GES_Lipid-EXT_ x Edu^#^ (low) |  | -0.13 (-1.65; 1.45) | 0.887 |  | -2.42 (-5.71; 1.18) | 0.188 |  | -2.05 (-5.74; 1.65) | 0.280 |
| GES_Lipid-EXT_ x Edu^#^ |  | 0.26 (-0.73; 1.25) | 0.630 |  | 0.01 (-2.30; 2.36) | 0.993 |  | -0.396 (-2.84; 2.11) | 0.772 |
| GES_Lipid-EXT_ x Edu^#^ (high) |  | ref. | - |  | ref. | - |  | ref. | - |
| Lipid ~ age + sex + GES_Lipid-EXT_ + Income + GES_Lipid-EXT_*Income | | | | | | | | | |
| Intercept | 4232 | -32.20 (-49.02; -15.39) | 1.8*10^-04^ | 4220 | 39.60 (8.64; 70.57) | 0.022 | 4233 | 54.55 (15.55; 93.55) | 6.1*10^-03^ |
| Age |  | 0.03 (-0.04; 0.09) | 0.416 |  | 0.28 (0.14; 0.42) | 8.9*10^-05^ |  | 0.36 (0.21; 0.51) | 2.2*10^-06^ |
| Sex |  | 14.10 (13.17; 15.04) | 1.4*10^-174^ |  | 0.03 (-2.12; 2.19) | 0.977 |  | 7.46 (5.16; 9.76) | 2.1*10^-10^ |
| GES_Lipid-EXT_ |  | 3.30 (2.51; 4.09) | 3.9*10^-16^ |  | 5.49 (3.60; 7.38) | 9.1*10^-09^ |  | 7.39 (5.41; 9.37) | 3.6*10^-13^ |
| Income (low) |  | 5.72 (-17.40; 28.83) | 0.628 |  | 3.59 (-37.43; 44.60) | 0.864 |  | 17.36 (-35.23; 39.95) | 0.518 |
| Income |  | 6.58 (-17.63; 30.79) | 0.594 |  | -2.02 (-45.50; 41.67) | 0.928 |  | 10.58 (-44.36; 65.83) | 0.70 |
| Income (high) |  | ref. | - |  | ref. | - |  | ref. | - |
| GES_Lipid-EXT_ x Inc^$^ (low) |  | -0.33 (-1.45; 0.79) | 0.562 |  | 0.00 (-2.60; 3.01) | 0.997 |  | -0.79 (-3.56; 1.98) | 0.569 |
| GES_Lipid-EXT_ x Inc^$^ |  | -0.20 (-1.58; 0.79) | 0.513 |  | 0.24 (-2.48; 3.01) | 0.850 |  | -0.53 (-3.43; 2.31) | 0.716 |
| GES_Lipid-EXT_ x Inc^$^ (high) |  | ref. | - |  | ref. | - |  | ref. | - |

^#^Education, ^$^Income

**Table S8**: Sex- and age-adjusted effects and corresponding 95% confidence intervals (95 % CI) on high-density lipoprotein cholesterol (HDL-C) in linear regression models of the joint effects of tertiles of a HDL-C-associated genetic effect allele sum score (GES_HDL-C-EXT_) based on [14-17] and socioeconomic position indicators, calculated separately for education groups and income tertiles, with the group of having a high GES_HDL-C-EXT_ and the highest socioeconomic position as reference.

| **Education** | | | |
| --- | --- | --- | --- |
|  | **n** | **ß (95% CI)** | ***p*** |
| ≤ 10 years | | | |
| Low GES_HDL-C-EXT_ | 171 | -10.66 (-13.42; -7.90) | 4.8*10^-14^ |
| Middle GES_HDL-C-EXT_ | 165 | -7.84 (-10.64; -5.04) | 4.2*10^-08^ |
| High GES_HDL-C-EXT_ | 179 | -5.17 (-7.89; -2.46) | 1.9*10^-04^ |
| 11-13 years | | | |
| Low GES_HDL-C-EXT_ | 835 | -9.24 (-11.01; -7.48) | 1.8*10^-24^ |
| Middle GES_HDL-C-EXT_ | 832 | -4.91 (-6.67; -3.14) | 5.3*10^-08^ |
| High GES_HDL-C-EXT_ | 835 | -2.28 (-4.04; -0.52) | 0.011 |
| ≥ 14 years | | | |
| Low GES_HDL-C-EXT_ | 493 | -7.04 (-8.99; -5.09) | 1.6*10^-12^ |
| Middle GES_HDL-C-EXT_ | 512 | -3.96 (-5.89; -2.03) | 6.0*10^-05^ |
| High GES_HDL-C-EXT_ | 481 | ref. | - |
| **Income** | | | |
|  | **n** | **ß (95% CI)** | ***p*** |
| Lower Tertile | | | |
| Low GES_HDL-C-EXT_ | 488 | -8.69 (-10.66; -6.73) | 5.5*10^-18^ |
| Middle GES_HDL-C-EXT_ | 527 | -4.97 (-6.90; -3.04) | 4.5*10^-07^ |
| High GES_HDL-C-EXT_ | 533 | -1.94 (-3.86; -0.01) | 0.049 |
| Middle Tertile | | | |
| Low GES_HDL-C-EXT_ | 424 | -8.85 (-10.89; -6.83) | 1.9*10^-17^ |
| Middle GES_HDL-C-EXT_ | 424 | -4.82 (-6.86; -2.79) | 3.4*10^-06^ |
| High GES_HDL-C-EXT_ | 391 | -2.78 (-4.86; -0.70) | 8.8*10^-03^ |
| Highest Tertile | | | |
| Low GES_HDL-C-EXT_ | 496 | -7.80 (-9.75; -5.85) | 5.9*10^-15^ |
| Middle GES_HDL-C-EXT_ | 472 | -4.12 (-6.09; -2.14) | 4.5*10^-05^ |
| High GES_HDL-C-EXT_ | 478 | ref. | - |

**Table S9**: Sex- and age-adjusted effects and corresponding 95% confidence intervals (95 % CI) on low-density lipoprotein cholesterol (LDL-C) and total cholesterol (TC) in linear regression models of the joint effects of tertiles of a LDL-C- and TC-associated genetic effect allele sum score (GES_LDL-C-EXT_ and GES_TC-EXT_) based on [14-17] and socioeconomic position indicators, calculated separately for education groups and income tertiles, with the group of having a low GES_LDL-C-EXT_ and GES_TC-EXT_ and the highest socioeconomic position as reference.

| LDL-C | | | | TC | | | | |  |
| --- | --- | --- | --- | --- | --- | --- | --- | --- | --- |
| **Education** | | | | | | | | |  |
|  | **n** | **β (95%-CI)** | ***p*** | **n** | **β (95%-CI)** | | ***p*** | | |
| ≤ 10 years | | | | | | | | |  |
| High GES_Lipid-EXT_ | 174 | 15.09 (8.73; 21.46) | 3.5*10^-06^ | 178 | | 15.69 (8.92; 22.46) | | 5.7*10^-06^ |  |
| Middle GES_Lipid-EXT_ | 177 | 11.08 (4.69; 17.46) | 6.8*10^-04^ | 174 | | 14.30 (7.50; 21.10) | | 3.8*10^-05^ |  |
| Low GES_Lipid-EXT_ | 164 | 5.57 (-0.96; 12.10) | 0.095 | 163 | | 5.30 (-1.64; 12.23) | | 0.134 |  |
| 11-13 years | | | | | | | | |  |
| High GES_Lipid-EXT_ | 809 | 15.42 (11.27; 19.57) | 3.8*10^-13^ | 827 | | 16.70 (12.33; 21.07) | | 8.2*10^-14^ |  |
| Middle GES_Lipid-EXT_ | 820 | 9.59 (5.44; 13.75) | 6.2*10^-06^ | 832 | | 11.55 (7.21; 15.90) | | 2.0*10^-07^ |  |
| Low GES_Lipid-EXT_ | 873 | 3.63 (-0.46; 7.72) | 0.082 | 843 | | 0.87 (-3.47; 5.20) | | 0.695 |  |
| ≥ 14 years | | | | | | | | |  |
| High GES_Lipid-EXT_ | 518 | 12.12 (7.62; 16.62) | 1.3*10^-07^ | 494 | | 15.39 (10.59; 20.19) | | 3.5*10^-10^ |  |
| Middle GES_Lipid-EXT_ | 504 | 6.52 (2.00; 11.05) | 4.7*10^-03^ | 500 | | 9.37 (4.58; 14.15) | | 1.3*10^-04^ |  |
| Low GES_Lipid-EXT_ | 464 | ref. | - | 492 | | ref. | | - |  |
| **Income** | | | | | | | | |  |
| Lower Tertile | | | | | | | | |  |
| High GES_Lipid-EXT_ | 503 | 13.39 (8.97; 17.81) | 3.0*10^-09^ | 524 | | 17.60 (12.93; 22.27) | | 1.7*10^-13^ |  |
| Middle GES_Lipid-EXT_ | 527 | 8.69 (4.31; 13.08) | 1.0*10^-04^ | 516 | | 14.30 (9.62; 18.97) | | 2.3*10^-09^ |  |
| Low GES_Lipid-EXT_ | 518 | 3.46 (-0.93; 7.84) | 0.122 | 508 | | 4.07 (-0.62; 8.76) | | 0.089 |  |
| Middle Tertile | | | | | | | | |  |
| High GES_Lipid-EXT_ | 442 | 13.16 (8.61; 17.72) | 1.6*10^-08^ | 441 | | 18.25 (13.38; 23.11) | | 2.3*10^-13^ |  |
| Middle GES_Lipid-EXT_ | 425 | 7.50 (2.89; 12.11) | 1.5*10^-03^ | 405 | | 9.75 (4.77; 14.73) | | 1.3*10^-04^ |  |
| Low GES_Lipid-EXT_ | 372 | -0.04 (-4.83; 4.75) | 0.988 | 393 | | 2.87 (-2.14; 7.89) | | 0.261 |  |
| Highest Tertile | | | | | | | | |  |
| High GES_Lipid-EXT_ | 459 | 9.88 (5.37; 14.39) | 1.8*10^-05^ | 438 | | 15.67 (10.81; 20.53) | | 2.9*10^-10^ |  |
| Middle GES_Lipid-EXT_ | 472 | 4.41 (-0.07; 8.88) | 0.054 | 498 | | 13.55 (8.85; 18.25) | | 1.7*10^-08^ |  |
| Low GES_Lipid-EXT_ | 515 | ref. | - | 510 | | ref. | | - |  |

**Table S10:** Sex- and age-adjusted effect size estimates and corresponding 95% confidence interval (95% CI) for the interaction of each LDL-C-increasing single-nucleotide polymorphism (SNP) based on [14] with low education on LDL-C (high education as reference), sorted by ascending effect size estimates.

| **Chr:Position*** | **Locus** | **SNP** | **Effect Allele** | **ß_SNPxEdu_** | **95 % CI** | | ***P*** |
| --- | --- | --- | --- | --- | --- | --- | --- |
|  |  |  |  |  | **Lower** | **Upper** |  |
| 1:55504650 | *PCSK9* | rs2479409 | G | -8.60 | -13.90 | -3.30 | 0.001 |
| 20:17845921 | *SNX5* | rs2328223 | C | -7.83 | -14.23 | -1.43 | 0.017 |
| 9:135144821 | *ST3GAL4* | rs11220462 | A | -6.51 | -13.97 | 0.95 | 0.087 |
| 6:16127407 | *MYLIP* | rs3757354 | C | -5.99 | -12.32 | 0.34 | 0.064 |
| 20:38524901 | *MAFB* | rs2902940 | A | -5.72 | -11.38 | -0.06 | 0.048 |
| 1:25775733 | *MACO1* | rs12027135 | T | -5.43 | -10.52 | -0.35 | 0.036 |
| 17:7091650 | *DLG4* | rs314253 | T | -5.22 | -10.61 | 0.17 | 0.058 |
| 8:55421614 | *RP1* | rs10102164 | A | -4.58 | -11.28 | 2.13 | 0.181 |
| 1:27138393 | *PIGV-NR0B2* | rs12748152 | T | -3.56 | -12.85 | 5.73 | 0.452 |
| 12:121416650 | *HNF1A* | rs1169288 | C | -3.21 | -8.63 | 2.21 | 0.245 |
| 8:59353534 | *UBXN2B* | rs13277801† | C | -2.85 | -8.25 | 2.56 | 0.303 |
| 8:9183358 | *LOC157273* | rs9987289 | G | -2.51 | -11.78 | 6.76 | 0.596 |
| 5:122855416 | *CSNK1G3* | rs4530754 | G | -2.47 | -7.76 | 2.83 | 0.361 |
| 2:234679384 | *UGT1A* | rs11563251 | T | -2.10 | -11.14 | 6.94 | 0.649 |
| 1:62798530 | *ANGPTL3* | rs2131925 | T | -1.98 | -7.70 | 3.74 | 0.497 |
| 7:44579180 | *NPC1L1* | rs2072183 | C | -1.78 | -7.69 | 4.12 | 0.554 |
| 16:56993324 | *CETP* | rs3764261 | A | -1.70 | -7.20 | 3.81 | 0.546 |
| 7:25991826 | *LOC105375199* | rs4722551 | C | -1.52 | -8.68 | 5.63 | 0.677 |
| 2:216304384 | *FN1* | rs1250229 | C | -1.46 | -7.39 | 4.46 | 0.629 |
| 12:112072424 | *BRAP* | rs11065987 | G | -1.27 | -6.43 | 3.89 | 0.630 |
| 2:121309488 | *LOC105373585* | rs2030746 | T | -1.26 | -6.50 | 3.98 | 0.638 |
| 6:32412435 | *HLA-DRA* | rs3177928 | A | -1.25 | -8.19 | 5.68 | 0.723 |
| 1:234853406 | none | rs558971† | G | -1.07 | -6.14 | 4.00 | 0.679 |
| 13:32953388 | *BRCA2* | rs4942486 | T | -1.00 | -6.14 | 4.14 | 0.704 |
| 20:12962718 | *SPTLC3* | rs364585 | A | -0.96 | -6.23 | 4.32 | 0.722 |
| 4:3473139 | *DOK7* | rs6831256 | G | -0.74 | -5.77 | 4.29 | 0.772 |
| 8:126490972 | *TRIB1* | rs2954029 | A | -0.56 | -5.58 | 4.45 | 0.827 |
| 3:32533010 | *CMTM6* | rs7640978 | T | -0.42 | -9.97 | 9.13 | 0.931 |
| 6:160578860 | *SLC22A1* | rs1564348 | C | 0.19 | -6.78 | 7.15 | 0.957 |
| 1:109619829 | *SORT1* | rs629301 | T | 0.25 | -6.13 | 6.64 | 0.939 |
| 7:21607352 | *DNAH11* | rs12670798 | C | 0.28 | -5.78 | 6.35 | 0.927 |
| 9:2640759 | *VLDLR* | rs3780181 | G | 0.36 | -9.83 | 10.55 | 0.945 |
| 16:72108093 | *HPR* | rs2000999 | A | 0.53 | -5.69 | 6.75 | 0.867 |
| 17:45438952 | *EFCAB13* | rs6504872† | T | 0.67 | -4.34 | 5.68 | 0.793 |
| 8:145022657 | *PLEC* | rs7832643† | T | 0.71 | -4.70 | 6.11 | 0.798 |
| 10:113933886 | *GPAM* | rs2255141 | A | 0.85 | -4.67 | 6.38 | 0.762 |
| 2:21263900 | *APOB* | rs1367117 | A | 0.99 | -4.75 | 6.73 | 0.736 |
| 1:220973563 | *MTARC1* | rs2642442 | C | 1.07 | -4.41 | 6.55 | 0.702 |
| 3:132163200 | *ACAD11* | rs17404153 | G | 1.16 | -6.50 | 8.82 | 0.766 |
| 20:39724338 | *TOP1* | rs6065311† | C | 1.19 | -3.97 | 6.36 | 0.652 |
| 19:11202306 | *LDLR* | rs6511720 | G | 1.34 | -7.03 | 9.72 | 0.754 |
| 1:150958836 | *ANXA9* | rs267733 | G | 1.40 | -5.80 | 8.61 | 0.703 |
| 5:156390297 | *TIMD4* | rs6882076 | T | 1.91 | -3.39 | 7.21 | 0.481 |
| 22:46627603 | *PPARA* | rs4253772 | T | 2.05 | -5.94 | 10.03 | 0.616 |
| 9:135144821 | *ST3GAL4* | rs11220462 | T | 2.26 | -4.03 | 8.55 | 0.481 |
| 19:45422946 | *APOE* | rs4420638 | G | 2.44 | -4.63 | 9.51 | 0.498 |
| 5:74656539 | *HMGCR* | rs12916 | C | 2.54 | -2.79 | 7.87 | 0.350 |
| 2:63149557 | *EHBP1* | rs2710642 | G | 2.74 | -2.54 | 8.03 | 0.309 |
| 22:30378703 | *MTMR3* | rs5763662 | T | 3.17 | -13.55 | 19.90 | 0.710 |
| 11:61569830 | *FADS1* | rs174546 | T | 3.24 | -2.13 | 8.61 | 0.237 |
| 2:44072576 | *ABCG5/8* | rs4299376 | G | 3.96 | -1.60 | 9.51 | 0.163 |
| 6:26093141 | *HFE* | rs1800562 | A | 3.97 | -8.66 | 16.61 | 0.538 |
| 14:24883887 | *NYNRIN* | rs8017377 | A | 4.27 | -0.85 | 9.40 | 0.102 |
| 6:116309649 | *FRK* | rs3798236† | C | 5.05 | -0.57 | 10.66 | 0.078 |
| 19:19407718 | *CILP2* | rs10401969 | C | 6.00 | -4.04 | 16.03 | 0.241 |
| 11:116648917 | ZPR1 | rs964184 | G | 6.46 | -0.53 | 6.37 | 0.070 |
| 2:118835841 | *INSIG2* | rs10490626 | G | 7.31 | -2.83 | 17.45 | 0.158 |
| 17:64210580 | *APOH-PRXCA* | rs1801689 | C | 8.28 | -5.51 | 22.07 | 0.239 |

*Position of build 37; †proxy SNPs (rs3798236 LD r²>1.0, rs7832643 LD r²>0.805, rs6504872 LD r²>0.935, rs6065311 LD r²>1.0, rs558971 LD r²>0.967 and rs13277801 LD r²>0.845) referenced on phase 3 haplotype data from the 1000 Genomes Project.

**Table S11:** Sex- and age-adjusted effect size estimates and corresponding 95% confidence interval (95% CI) for the interaction of each HDL-C-increasing single-nucleotide polymorphism (SNP) based on [14] with low education on HDL-C (high education as reference), sorted by ascending effect size estimates.

| **Chr:Position*** | **Locus** | **SNP** | **Effect Allele** | **ß_SNPxEdu_** | **95 % CI** | | ***P*** |
| --- | --- | --- | --- | --- | --- | --- | --- |
|  |  |  |  |  | **Lower** | **Upper** |  |
| 11:51512090 | *OR4C46* | rs11246602 | C | -4.98 | -8.57 | -1.39 | 0.007 |
| 8:19844222 | *LPL* | rs12678919 | G | -4.11 | -8.09 | -0.12 | 0.043 |
| 20:43042364 | *HNF4A* | rs1800961 | C | -4.03 | -11.26 | 3.21 | 0.275 |
| 19:54792761 | *LILRA3* | rs386000 | C | -3.17 | -6.02 | -0.32 | 0.029 |
| 4:103188709 | *SLC39A8* | rs13107325 | C | -2.72 | -7.51 | 2.06 | 0.264 |
| 8:9183358 | *PPP1R3B* | rs9987289 | A | -2.50 | -6.53 | 1.53 | 0.225 |
| 12:20473758 | *PDE3A* | rs7134375 | A | -2.13 | -4.46 | 0.20 | 0.073 |
| 15:58683366 | *LIPC* | rs1532085 | A | -2.09 | -4.38 | 0.20 | 0.073 |
| 11:46743247 | *LRP4* | rs3136441 | C | -1.97 | -5.20 | 1.25 | 0.230 |
| 7:6449272 | *DAGLB* | rs702485 | G | -1.90 | -4.20 | 0.40 | 0.105 |
| 18:47159090 | *LOC105372112* | rs7240405† | A | -1.84 | -4.93 | 1.24 | 0.242 |
| 7:17919258 | *SNX13* | rs4142995 | T | -1.76 | -4.06 | 0.55 | 0.136 |
| 2:226808942 | *IRS1* | rs2972146 | G | -1.70 | -4.01 | 0.61 | 0.149 |
| 15:63396867 | *LACTB* | rs2652834 | G | -1.70 | -4.29 | 0.90 | 0.200 |
| 11:75455021 | *MOGAT2-DGAT2* | rs499974 | C | -1.55 | -4.31 | 1.21 | 0.271 |
| 9:107664301 | *ABCA1* | rs1883025 | T | -1.49 | -4.05 | 1.08 | 0.256 |
| 2:57792580 | *LRP1* | rs11613352 | T | -1.25 | -3.79 | 1.29 | 0.336 |
| 1:182168885 | *ZNF648* | rs1689800 | A | -1.00 | -3.32 | 1.33 | 0.402 |
| 16:56993324 | *CETP* | rs3764261 | A | -0.82 | -3.20 | 1.55 | 0.496 |
| 8:126490972 | *TRIB1* | rs2954029 | T | -0.71 | -2.89 | 1.47 | 0.524 |
| 6:139831757 | none | rs634869† | T | -0.70 | -2.98 | 1.58 | 0.546 |
| 19:33899065 | *PEPD* | rs731839 | G | -0.54 | -2.93 | 1.85 | 0.659 |
| 20:44554015 | *PLTP* | rs6065906 | C | -0.48 | -3.28 | 2.33 | 0.740 |
| 19:11347493 | *ANGPTL8* | rs737337 | T | -0.38 | -4.42 | 3.66 | 0.853 |
| 3:52532118 | *STAB1* | rs13326165 | A | -0.38 | -3.13 | 2.37 | 0.787 |
| 4:89741269 | *FAM13A* | rs3822072 | G | -0.33 | -2.55 | 1.89 | 0.774 |
| 11:116648917 | *APOA1* | rs964184 | C | -0.32 | -3.35 | 2.70 | 0.834 |
| 12:109938857 | *UBE3B* | rs2241213† | G | -0.25 | -2.51 | 2.00 | 0.825 |
| 19:8431581 | *ANGPTL4* | rs2278236† | A | -0.22 | -2.49 | 2.04 | 0.846 |
| 4:26062990 | *C4orf52* | rs10019888 | G | -0.20 | -3.26 | 2.87 | 0.900 |
| 11:10388782 | *AMPD3* | rs2923084 | A | -0.12 | -2.98 | 2.73 | 0.932 |
| 14:105277209 | *ZBTB42-AKT1* | rs4983559 | G | -0.11 | -2.35 | 2.14 | 0.927 |
| 11:65391317 | *KAT5* | rs12801636 | A | -0.10 | -2.80 | 2.60 | 0.941 |
| 5:53298025 | *ARL15* | rs6450176 | A | -0.01 | -2.53 | 2.50 | 0.991 |
| 3:50129399 | *RBM5* | rs2013208 | T | 0.00 | -2.31 | 2.32 | 0.999 |
| 2:211540507 | *CPS1* | rs1047891 | C | 0.08 | -2.30 | 2.47 | 0.947 |
| 16:81534790 | *CMIP* | rs2925979 | C | 0.11 | -2.32 | 2.53 | 0.932 |
| 11:61569830 | *FADS1-2-3* | rs174546 | T | 0.11 | -2.23 | 2.45 | 0.926 |
| 1:156700651 | *HDGF-PMVK* | rs12145743 | G | 0.14 | -2.24 | 2.52 | 0.909 |
| 17:76403984 | *PGS1* | rs4129767 | A | 0.16 | -2.08 | 2.39 | 0.890 |
| 11:122522375 | *UBASH3B* | rs7941030 | C | 0.17 | -2.13 | 2.47 | 0.886 |
| 17:35067382 | *STARD3* | rs11869286 | G | 0.21 | -2.24 | 2.66 | 0.868 |
| 7:150529449 | *TMEM176A* | rs17173637 | T | 0.32 | -3.17 | 3.80 | 0.858 |
| 9:15295378 | *TTC39B* | rs581080 | C | 0.38 | -2.51 | 3.28 | 0.795 |
| 7:50305863 | *IKZF1* | rs4917014 | G | 0.40 | -1.95 | 2.76 | 0.739 |
| 12:124481690 | *ZNF664* | rs7975482† | G | 0.41 | -1.87 | 2.70 | 0.723 |
| 1:230295691 | *GALNT2* | rs4846914 | G | 0.46 | -1.81 | 2.73 | 0.691 |
| 10:46013277 | *MARCH8-ALOX5* | rs970548 | C | 0.59 | -1.98 | 3.15 | 0.654 |
| 1:40028180 | *PABPC4* | rs4660293 | G | 0.59 | -2.01 | 3.19 | 0.657 |
| 18:56000003 | *MC4R* | rs12967135 | G | 0.61 | -1.96 | 3.19 | 0.641 |
| 6:127450254 | *RSPO3* | rs1936800 | C | 0.66 | -1.58 | 2.90 | 0.564 |
| 1:178515312 | *ANGPTL1* | rs4650994 | G | 0.74 | -1.46 | 2.93 | 0.511 |
| 19:45422946 | *APOE* | rs4420638 | A | 0.77 | -2.32 | 2.58 | 0.626 |
| 3:119560606 | *GSK3B* | rs6805251 | T | 0.78 | -1.45 | 3.01 | 0.493 |
| 2:165540800 | *COBLL1* | rs12328675 | C | 0.82 | -2.33 | 3.97 | 0.610 |
| 3:47061183 | *SETD2* | rs2290547 | A | 0.86 | -2.04 | 3.76 | 0.562 |
| 16:67928042 | *LCAT* | rs16942887 | A | 0.92 | -2.32 | 4.16 | 0.578 |
| 7:130433384 | *KLF14* | rs4731702 | T | 0.93 | -1.30 | 3.16 | 0.413 |
| 4:100014805 | *ADH5* | rs2602836 | A | 1.01 | -1.24 | 3.26 | 0.378 |
| 3:132163200 | *ACAD11* | rs17404153 | T | 1.15 | -2.19 | 4.48 | 0.501 |
| 3:11400249 | *ATG7* | rs2606736 | C | 1.16 | -1.15 | 3.47 | 0.325 |
| 17:64386889 | *ABCA8* | rs4148008 | G | 1.23 | -1.20 | 3.66 | 0.320 |
| 16:53809247 | *FTO* | rs1121980 | A | 1.46 | -0.77 | 3.69 | 0.199 |
| 19:52324216 | *HAS1* | rs17695224 | A | 1.67 | -0.87 | 4.22 | 0.197 |
| 8:116599199 | *TRPS1* | rs2293889 | G | 1.74 | -0.52 | 4.00 | 0.131 |
| 7:72982874 | *MLXIPL* | rs17145738 | T | 1.77 | -1.81 | 5.34 | 0.333 |
| 22:21932068 | *UBE2L3* | rs181362 | C | 1.91 | -0.81 | 4.63 | 0.169 |
| 6:43865874 | *VEGFA* | rs998584 | A | 2.00 | -0.28 | 4.28 | 0.086 |
| 12:125261593 | *SCARB1* | rs838880 | C | 2.74 | 0.33 | 5.15 | 0.026 |
| 12:123775127 | *SBNO1* | rs10773003† | A | 2.77 | -1.12 | 6.65 | 0.163 |
| 1:27138393 | *PIGV-NR0B2* | rs12748152 | C | 3.00 | -1.03 | 7.02 | 0.145 |

*Position of build 37; †proxy SNPs (rs634869 LD r²>1.0, rs10773003 LD r²>1.0, rs7975482 LD r²>1.0, rs2241213 LD r²>0.935, rs7240405 LD r²>1.0 and rs2278236 LD r²>0.965) referenced on phase 3 haplotype data from the 1000 Genomes Project.

**Table S12:** Sex- and age-adjusted effect size estimates and corresponding 95% confidence interval (95% CI) for the interaction of each TC-increasing single-nucleotide polymorphism (SNP) based on [14] with low education on TC (high education as reference), sorted by ascending effect size estimates.

| **Chr:Position*** | **Locus** | **SNP** | **Effect Allele** | **ß_SNPxEdu_** | **95 % CI** | | ***P*** |
| --- | --- | --- | --- | --- | --- | --- | --- |
|  |  |  |  |  | **Lower** | **Upper** |  |
| 2:118835841 | *INSIG2* | rs10490626 | A | -9.10 | -20.01 | 1.81 | 0.102 |
| 1:55504650 | *PCSK9* | rs2479409 | G | -8.29 | -14.00 | -2.59 | 0.004 |
| 2:234679384 | *UGT1A* | rs11563251 | T | -7.28 | -16.99 | 2.45 | 0.143 |
| 3:12674199 | *RAF1* | rs11709504† | T | -7.25 | -14.35 | -0.14 | 0.046 |
| 2:203532304 | *FAM117B* | rs11694172 | G | -6.80 | -13.31 | -0.30 | 0.041 |
| 20:38524901 | *MAFB* | rs2902940 | A | -6.75 | -12.80 | -0.71 | 0.029 |
| 9:135144821 | *ST3GAL4* | rs11220462 | A | -6.72 | -14.75 | 1.31 | 0.101 |
| 19:49206417 | *FLJ36070* | rs492602 | G | -5.85 | -11.38 | -0.32 | 0.038 |
| 1:62798530 | *ANGPTL3* | rs2131925 | T | -5.05 | -11.19 | 1.09 | 0.107 |
| 2:121309488 | *LOC105373585* | rs2030746 | T | -4.98 | -10.61 | 0.66 | 0.084 |
| 1:23766233 | *ASAP3* | rs1077514 | T | -4.87 | -12.55 | 2.81 | 0.214 |
| 9:15295378 | *TTC39B* | rs581080 | G | -4.77 | -11.93 | 2.39 | 0.192 |
| 7:44579180 | *NPC1L1* | rs2072183 | C | -4.77 | -11.11 | 1.58 | 0.141 |
| 22:35711098 | *TOM1* | rs138777 | A | -4.30 | -10.15 | 1.55 | 0.150 |
| 17:7091650 | *DLG4* | rs314253 | T | -3.52 | -9.31 | 2.27 | 0.233 |
| 8:55421614 | *RP1* | rs10102164 | A | -3.19 | -10.40 | 4.03 | 0.387 |
| 9:2640759 | *VLDLR* | rs3780181 | A | -2.90 | -13.65 | 7.85 | 0.597 |
| 1:2348534061 | none | rs558971† | G | -2.77 | -8.22 | 2.68 | 0.319 |
| 10:46013277 | *MARCH8-ALOX5* | rs970548 | C | -2.25 | -8.59 | 4.09 | 0.487 |
| 8:126490972 | *TRIB1* | rs2954029 | A | -1.69 | -7.08 | 3.71 | 0.541 |
| 2:135837906 | *RAB3GAP1* | rs7570971 | A | -1.64 | -7.13 | 3.85 | 0.558 |
| 1:109619829 | *SORT1* | rs629301 | T | -1.63 | -8.50 | 5.24 | 0.643 |
| 16:56993324 | *CETP* | rs3764261 | A | -1.46 | -7.38 | 4.45 | 0.628 |
| 8:182724388 | none | rs4921914† | C | -1.39 | -8.00 | 5.22 | 0.680 |
| 7:21607352 | *DNAH11* | rs12670798 | C | -1.21 | -7.72 | 5.31 | 0.716 |
| 12:9082581 | *PHC1-A2ML1* | rs4883201 | A | -1.19 | -9.75 | 7.37 | 0.786 |
| 8:59353534 | *UBXN2B* | rs13277801† | C | -1.09 | -6.90 | 4.73 | 0.715 |
| 12:112072424 | *BRAP* | rs11065987 | G | -1.05 | -6.59 | 4.49 | 0.710 |
| 5:156390297 | *TIMD4* | rs6882076 | C | -1.04 | -6.73 | 4.66 | 0.721 |
| 2:21263900 | *APOB* | rs1367117 | A | -0.89 | -7.04 | 5.26 | 0.777 |
| 11:18632984 | *SPTY2D1* | rs10128711 | T | -0.84 | -7.04 | 5.36 | 0.790 |
| 12:121416650 | *HNF1A* | rs1169288 | C | -0.82 | -6.65 | 5.00 | 0.781 |
| 17:45438952 | *EFCAB13* | rs6504872† | T | -0.64 | -6.03 | 4.75 | 0.816 |
| 11:118486067 | *PHLDB1* | rs11603023 | T | -0.58 | -6.33 | 5.17 | 0.843 |
| 8:9183358 | *LOC157273* | rs9987289 | A | -0.55 | -10.52 | 9.42 | 0.914 |
| 7:1083777 | *GPR146* | rs1997243 | G | -0.34 | -7.68 | 7.01 | 0.929 |
| 15:58683366 | *LIPC* | rs1532085 | A | -0.32 | -5.99 | 5.36 | 0.913 |
| 19:45422946 | *APOE* | rs4420638 | G | -0.22 | -7.84 | 7.40 | 0.954 |
| 22:46627603 | *PPARA* | rs4253772 | T | -0.20 | -8.79 | 8.39 | 0.964 |
| 4:3473139 | *DOK7* | rs6831256 | G | -0.16 | -5.58 | 5.25 | 0.953 |
| 6:32412435 | *HLA-DRA* | rs3177928 | A | -0.12 | -7.57 | 7.34 | 0.976 |
| 7:25991826 | *LOC105375199* | rs4722551 | C | 0.14 | -7.54 | 7.81 | 0.972 |
| 3:32533010 | *CMTM6* | rs7640978 | T | 0.41 | -9.83 | 10.65 | 0.938 |
| 6:26093141 | *HFE* | rs1800562 | G | 0.56 | -13.03 | 14.16 | 0.935 |
| 16:72108093 | *HPR* | rs2000999 | A | 0.70 | -5.99 | 7.39 | 0.838 |
| 6:39250837 | *KCNK17* | rs2758886 | A | 0.73 | -5.28 | 6.75 | 0.811 |
| 10:113933886 | *GPAM* | rs2255141 | A | 0.99 | -4.95 | 6.93 | 0.743 |
| 11:61569830 | *FADS1* | rs174546 | T | 1.21 | -4.57 | 6.98 | 0.682 |
| 9:107664301 | *ABCA1* | rs1883025 | C | 1.32 | -5.03 | 7.66 | 0.685 |
| 11:122522375 | *UBASH3B* | rs7941030 | C | 1.48 | -4.21 | 7.17 | 0.609 |
| 20:39724338 | *TOP1* | rs6065311† | C | 1.51 | -4.04 | 7.06 | 0.594 |
| 6:34546560 | *C6orf106* | rs2814982 | C | 1.56 | -6.81 | 9.92 | 0.715 |
| 3:58381287 | *PXK* | rs13315871 | A | 1.75 | -7.28 | 10.78 | 0.704 |
| 2:169830155 | *ABCB11* | rs2287623 | G | 1.86 | -3.99 | 7.70 | 0.534 |
| 1:220973563 | *MTARC1* | rs2642442 | C | 2.05 | -3.86 | 7.95 | 0.497 |
| 5:122855416 | *CSNK1G3* | rs4530754 | A | 2.18 | -3.51 | 7.87 | 0.452 |
| 5:74656539 | *HMGCR* | rs12916 | C | 2.57 | -3.17 | 8.30 | 0.380 |
| 6:160578860 | *SLC22A1* | rs1564348 | C | 2.71 | -4.77 | 10.20 | 0.478 |
| 11:116648917 | *ZPR1* | rs964184 | G | 2.91 | -4.59 | 10.42 | 0.447 |
| 2:44072576 | *ABCG5/8* | rs4299376 | G | 3.21 | -2.75 | 9.17 | 0.291 |
| 8:145022657 | *PLEC* | rs7832643† | T | 3.42 | -2.39 | 9.23 | 0.248 |
| 19:11202306 | *LDLR* | rs6511720 | G | 3.84 | -5.18 | 12.85 | 0.404 |
| 6:16127407 | *MYLIP* | rs3757354 | T | 4.21 | -2.60 | 11.01 | 0.225 |
| 1:25775733 | *MACO1* | rs12027135 | A | 4.52 | -0.95 | 9.99 | 0.105 |
| 9:135144821 | *ST3GAL4* | rs11220462 | T | 5.42 | -1.34 | 12.19 | 0.116 |
| 2:27730940 | *GCKR* | rs1260326 | T | 5.69 | 0.21 | 11.16 | 0.042 |
| 19:19407718 | *CILP2* | rs10401969 | C | 5.73 | -5.06 | 16.52 | 0.298 |
| 6:135376209 | *HBS1L* | rs9376090 | T | 5.74 | -0.65 | 12.13 | 0.078 |
| 1:92976590 | *EVI5* | rs6690764† | A | 6.22 | -0.64 | 13.08 | 0.076 |
| 6:116309649 | *FRK* | rs3798236† | C | 7.24 | 1.23 | 13.25 | 0.018 |
| 20:34152782 | *ERGIC3* | rs2277862 | T | 7.65 | -0.09 | 15.39 | 0.053 |
| 10:17260290 | *VIM-CUBN* | rs10904908 | G | 8.04 | 2.59 | 13.50 | 0.004 |
| 18:47159090 | *LOC105372112*† | rs7240405 | A | 9.36 | 1.72 | 17.01 | 0.016 |
| 20:43042364 | *HNF4A* | rs1800961 | C | 19.70 | 1.83 | 37.56 | 0.031 |

*Position of build 37; †proxy SNPs (rs7240405 LD r²>1.0, rs3798236 LD r²>1.0, rs6690764 LD r²>1.0, rs7832643 LD r²>0.805, rs6065311 LD r²>1.0, rs6504872 LD r²>0.935, rs13277801 LD r²>0.845, rs4921914 LD r²>1.0, rs11709504 LD r²>0.8, rs558971 LD r²>0.967) referenced on phase 3 haplotype data from the 1000 Genomes Project.

**Table S13:** Sensitivity analysis: Sex-, age- and lipid-lowering medication (LLM) adjusted effects per GES_Lipid_ standard deviation and corresponding 95% confidence intervals (95% CI) on high-density lipoprotein cholesterol (HDL-C), low-density lipoprotein cholesterol (LDL-C) and total cholesterol (TC) in linear regression models including main effects of education groups (≤10 years/11-13 years/≥14 years), genetic effect allele sum scores (GES_Lipid_) based on [14] and interaction terms of GES_Lipid_ and education.

| HDL-C | | | | LDL-C | | | TC | | |
| --- | --- | --- | --- | --- | --- | --- | --- | --- | --- |
| Lipid ~ Education + age + sex + lipid-lowering medication | | | | | | | | | |
|  | **n** | **β (95%-CI)** | ***p*** | **n** | **β (95%-CI)** | ***p*** | **n** | **β (95%-CI)** | ***p*** |
| Intercept | 4218 | 32.76 (28.80; 36.71) | 2.0*10^-57^ | 4205 | 125.04 (115.91; 134.17) | 1.1*10^-146^ | 4219 | 190.78 (180.96; 200.60) | 5.3*10^-273^ |
| Age |  | 0.08 (0.02; 0.14) | 8.4*10^-03^ |  | 0.38 (0.23; 0.52) | 2.2*10^-07^ |  | 0.47 (0.32; 0.63) | 1.4*10^-09^ |
| Sex |  | 14.75 (13.75; 15.74) | 1.8*10^-169^ |  | -0.90 (-3.19; 1.39) | 0.440 |  | 7.81 (5.34; 10.28) | 6.0*10^-10^ |
| LLM |  | -3.14 (-4.53; -1.74) | 1.1*10^-05^ |  | -20.58 (-23.80; -17.36) | 2.2*10^-35^ |  | -20.85 (-24.31; -17.39) | 1.1*10^-31^ |
| Education (low) |  | -4.31 (-6.02; -2.60) | 8.0*10^-07^ |  | 4.68 (0.74; 8.62) | 0.020 |  | 3.83 (-0.42; 8.07) | 0.077 |
| Education |  | -1.72 (-2.79; -0.65) | 1.7*10^-03^ |  | 2.70 (0.23; 5.17) | 0.032 |  | 0.90 (-1.76; 3.55) | 0.510 |
| Education (high) |  | ref. |  |  | ref. | - |  | ref. | - |
| Lipid ~ GES_Lipid_ + age + sex + lipid-lowering medication | | | | | | | | | |
| Intercept | 4226 | -9.95 (-18.00; -1.90) | 0.016 | 4213 | 37.96 (22.35; 53.57) | 1.9*10^-06^ | 4227 | 66.88 (48.01; 85.74) | 4.2*10^-12^ |
| Age |  | 0.05 (-0.01; 0.11) | 0.120 |  | 0.41 (0.28; 0.55) | 3.0*10^-09^ |  | 0.50 (0.35; 0.64) | 3.1*10^-11^ |
| Sex |  | 13.91 (12.99; 14.83) | 3.0*10^-175^ |  | -0.16 (-2.27; 1.95) | 0.880 |  | 7.90 (5.64; 10.16) | 8.0*10^-12^ |
| LLM |  | -3.09 (-4.47; -1.71) | 1.1*10^-05^ |  | -22.71 (25.89; -19.54) | 1.1*10^-43^ |  | -23.69 (-27.08; -20.29) | 1.2*10^-41^ |
| GES_Lipid_ |  | 2.91 (2.45; 3.37) | 5.9*10^-34^ |  | 7.02 (5.94; 8.06) | 3.0*10^-38^ |  | 8.48 (7.38; 9.62) | 9.0*10^-48^ |
| Lipid ~ Education + GES_Lipid_ + Education*GES_Lipid_ + age + sex + lipid-lowering medication | | | | | | | | | |
| Intercept | 4218 | -11.36 (-24.45; 1.73) | 0.089 | 4205 | 27.93 (2.84; 53.01) | 0.029 | 4219 | 55.68 (24.85; 86.51) | 4.0*10^-04^ |
| Age |  | 0.08 (0.02; 0.14) | 0.013 |  | 0.38 (0.24; 0.52) | 7.6*10^-08^ |  | 0.47 (0.32; 0.62) | 5.8*10^-10^ |
| Sex |  | 14.70 (13.72; 15.68) | 2.5*10^-173^ |  | -1.33 (-3.58; 0.91) | 0.245 |  | 7.19 (4.78; 9.59) | 5.3*10^-09^ |
| GES_Lipid_ |  | 2.91 (2.09; 3.72) | 4.8*10^-12^ |  | 8.01 (6.08; 9.90) | 3.3*10^-16^ |  | 9.41 (7.38; 11.44) | 9.7*10^-20^ |
| LLM |  | 1.25 (-19.31; 21.81) | 0.910 |  | 7.23 (-33.70; 48.17) | 0.729 |  | 15.13 (-36.23; 66.49) | 0.564 |
| Education (low) |  | -0.41 (-24.80; 23.98) | 0.974 |  | 50.45 (5.83; 95.07) | 0.027 |  | 49.62 (-7.45; 106.67) | 0.088 |
| Education |  | -3.37 (-19.01; 12.27) | 0.673 |  | 6.43 (-22.34; 35.20) | 0.661 |  | 5.41 (-30.93; 41.75) | 0.770 |
| Education (high) |  | ref. | - |  | ref. | - |  | ref. | - |
| GES_Lipid_ x Edu^#^ (low) |  | -0.25 (-1.84; 1.38) | 0.770 |  | -3.74 (-7.38; 0.09) | 0.045 |  | -3.17 (-7.07; 0.73) | 0.113 |
| GES_Lipid_ x Edu^#^ |  | 0.01 (-6.02; 1.12) | 0.844 |  | -0.36 (-2.70; 1.98) | 0.766 |  | -0.36 (-2.86; 2.13) | 0.768 |
| GES_Lipid_ x Edu^#^ (high) |  | ref. | - |  | ref. | - |  | ref. | - |
| LLM x Edu^#^ (low) |  | -1.32 (-5.73; 3.09) | 0.557 |  | 3.34 (-6.87; 13.56) | 0.521 |  | 4.38 (-6.60; 15.36) | 0.434 |
| LLM x Edu^#^ |  | 0.61 (-2.42; 3.64) | 0.693 |  | 8.18 (1.20; 15.17) | 0.022 |  | 9.17 (1.70; 16.64) | 0.016 |
| LLM x Edu^#^ (high) |  | ref. | - |  | ref. | - |  | ref. | - |
| GES_Lipid_ x LLM |  | -0.31 (-1.63; 1.07) | 0.673 |  | -2.79 (-6.03; 0.45) | 0.091 |  | -3.02 (-6.45; 0.47) | 0.088 |

^#^Education

**Table S14**: Sensitivity analysis: Sex- and age- adjusted effects per GES_Lipid_ standard deviation and corresponding 95% confidence intervals (95% CI) on high-density lipoprotein cholesterol (HDL-C), low-density lipoprotein cholesterol (LDL-C) and total cholesterol (TC) in linear regression models including main effects of a lipid-associated genetic effect allele sum score (GES_Lipid_) based on [14], education and interaction terms of GES_Lipid_ and education a) adjusted for lipid-lowering medication and b) under exclusion of participants with lipid-lowering medication.

| HDL-C | | | | LDL-C | | | TC | | |
| --- | --- | --- | --- | --- | --- | --- | --- | --- | --- |
| Lipid ~ Education + age + sex (exclusion of participants on lipid lowering medication) | | | | | | | | | |
|  | **n** | **β (95%-CI)** | ***p*** | **n** | **β (95%-CI)** | ***p*** | **n** | **β (95%-CI)** | ***p*** |
| Intercept | 3945 | 34.21 (30.07; 38.35) | 3.0*10^-57^ | 3934 | 124.61 (115.21; 134.02) | 2.3*10^-137^ | 3946 | 190.83 (180.80; 200.86) | 4.5*10^-261^ |
| Age |  | 0.06 (-0.01; 0.12) | 0.089 |  | 0.43 (0.29; 0.58) | 7.0*10^-09^ |  | 0.54 (0.38; 0.69) | 1.6*10^-11^ |
| Sex |  | 15.04 (14.01; 16.08) | 1.1*10^-161^ |  | -2.93 (-5.29; -0.57) | 0.015 |  | 5.49 (2.98; 8.01) | 1.9*10^-05^ |
| Education (low) |  | -3.99 (-5.80; -2.17) | 1.6*10^-05^ |  | 4.78 (0.66; 8.90) | 0.023 |  | 3.61 (-0.78; 8.00) | 0.107 |
| Education |  | -1.96 (-3.08; -0.84) | 6.4*10^-04^ |  | 2.46 (-0.08; 5.01) | 0.058 |  | 0.59 (-2.13; 3.30) | 0.672 |
| Education (high) |  | ref. |  |  | ref. | - |  | ref. | - |
| Lipid ~ GES_Lipid_ + age + sex (exclusion of participants on lipid lowering medication) | | | | | | | | | |
| Intercept | 3958 | -9.28 (-17.78; -0.78) | 0.037 | 3947 | 34.60 (18.49; 50.71) | 2.6*10^-05^ | 3959 | 66.49 (47.13; 85.84) | 1.9*10^-11^ |
| Age |  | 0.02 (-0.04; 0.09) | 0.553 |  | 0.47 (0.33; 0.61) | 6.2*10^-11^ |  | 0.56 (0.41; 0.71) | 2.6*10^-13^ |
| Sex |  | 14.30 (13.33; 15.27) | 3.9*10^-167^ |  | -2.30 (-4.49; -0.11) | 0.040 |  | 5.43 (3.11; 7.75) | 4.7*10^-06^ |
| GES_Lipid_ |  | 2.91 (2.45; 3.42) | 7.5*10^-31^ |  | 7.29 (6.17; 8.37) | 2.3*10^-38^ |  | 8.53 (7.38; 9.72) | 9.2*10^-46^ |
| Lipid ~ Education + GES_Lipid_ + Education*GES_Lipid_ + age + sex (exclusion of participants on lipid lowering medication) | | | | | | | | | |
| Intercept | 3945 | -9.29 (-22.68; 4.09) | 0.174 | 3934 | 28.93 (3.53; 54.33) | 0.026 | 3946 | 54.22 (23.07; 85.36) | 6.5*10^-04^ |
| Age |  | 0.05 (-0.01; 0.12) | 0.098 |  | 0.44 (0.29; 0.58) | 2.3*10^-09^ |  | 0.54 (0.39; 0.69) | 5.2*10^-12^ |
| Sex |  | 14.98 (13.96; 16.00) | 2.7*10^-165^ |  | -3.42 (-5.74; -1.11) | 3.7*10^-03^ |  | 4.79 (2.34; 7.25) | 1.3*10^-04^ |
| GES_Lipid_ |  | 2.86 (1.99; 3.67) | 3.1*10^-11^ |  | 7.83 (5.90; 9.77) | 2.1*10^-15^ |  | 9.46 (7.44; 11.54) | 1.1*10^-19^ |
| Education (low) |  | 0.40 (-25.90; 26.71) | 0.976 |  | 50.37 (3.48; 97.26) | 0.035 |  | 43.89 (-14.70; 102.49) | 0.142 |
| Education |  | -4.95 (-21.39; 11.50) | 0.555 |  | 6.25 (-23.31; 35.81) | 0.679 |  | 16.91 (-20.42; 54.24) | 0.375 |
| Education (high) |  | ref. | - |  | ref. | - |  | ref. | - |
| GES_Lipid_ x Edu^#^ (low) |  | -0.26 (-1.99; 1.43) | 0.749 |  | -3.69 (-7.52; 0.17) | 0.060 |  | -2.76 (-6.76 ; 1.30) | 0.183 |
| GES_Lipid_ x Edu^#^ |  | 0.20 (-0.87; 1.28) | 0.719 |  | 0.27 (-2.70; 2.19) | 0.828 |  | -1.10 (-3.64 ; 1.46) | 0.401 |
| GES_Lipid_ x Edu^#^ (high) |  | ref. | - |  | ref. | - |  | ref. | - |

^#^Education

**study population**

**n=4,814**

**analysis population**

**n=4,516**

**HDL-C**

**n=4,515**

**LDL-C**

**n=4,502**

**TC**

**n=4,516**

no genetic information n=296

missing values for lipids n=22

no LDL-C n=14

no HDL-C n=1

**Figure S1:** Flowchart of participants of the Heinz Nixdorf Recall Study (HNR) cohort included in the analysis.

**a**

**c**

**ß GES_TC-EXT_**

**ß GES_HDL-C-EXT_**

**b**

**ß GES_LDL-C-EXT_**

**Figure S2** Sex-, age-adjusted and effects per GES_Lipid-EXT_ standard deviation and corresponding 95% confidence interval (95% CI) of the genetic effect on a) high-density lipoprotein cholesterol (HDL-C), b) low-density lipoprotein cholesterol (LDL-C) and c) total cholesterol (TC), stratified by education groups (years) and income tertiles in linear regression models using the GES_Lipid-EXT_ based on [14-17].


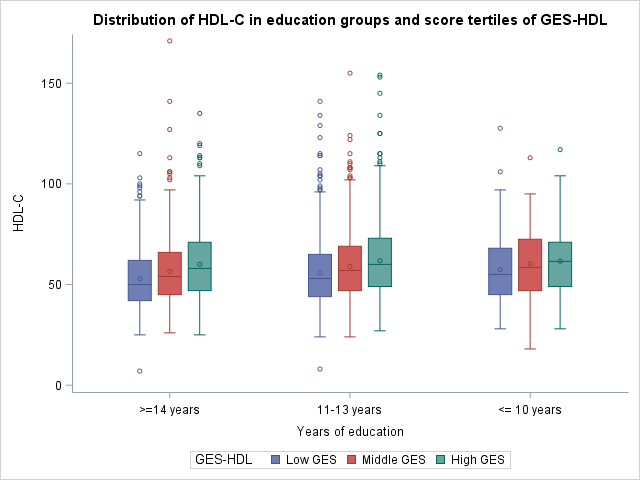

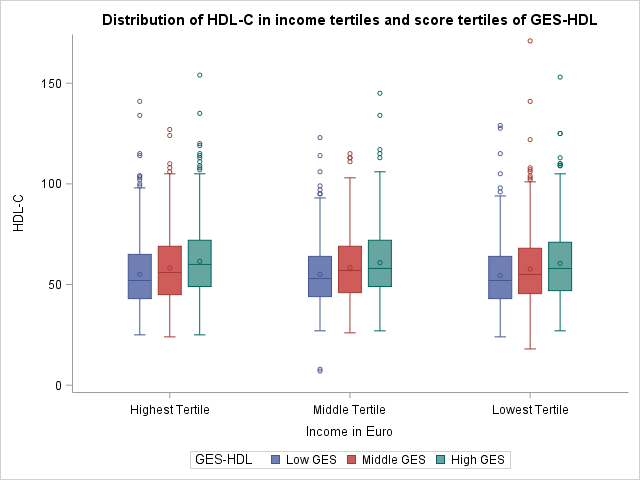


b

a

| **Income** | **GES_HDL-C_** | **N** | **Mean** | **Median** | **SD** | **Q1** | **Q3** | **Min** | **Max** |
| --- | --- | --- | --- | --- | --- | --- | --- | --- | --- |
| **Highest Tertile** | **Low GES** | 481 | 55.1 | 52 | 17.2 | 43 | 65 | 25 | 141 |
|  | **Middle GES** | 488 | 58.2 | 56 | 17.4 | 45 | 69 | 24 | 127 |
|  | **High GES** | 477 | 61.6 | 60 | 18.2 | 49 | 72 | 25 | 154 |
| **Middle Tertile** | **Low GES** | 444 | 55 | 53 | 15.7 | 44 | 64 | 7 | 123 |
|  | **Middle GES** | 373 | 58.4 | 57 | 16.2 | 46 | 69 | 26 | 115 |
|  | **High GES** | 421 | 60.9 | 58 | 17.4 | 49 | 72 | 27 | 145 |
| **Lowest Tertile** | **Low GES** | 489 | 54.5 | 52 | 15.9 | 43 | 64 | 24 | 129 |
|  | **Middle GES** | 548 | 57.6 | 55 | 17.6 | 45.5 | 68 | 18 | 171 |
|  | **High GES** | 511 | 60.5 | 58 | 17.8 | 47 | 71 | 27 | 153 |

| **Education** | **GES_HDL-C_** | **N** | **Mean** | **Median** | **SD** | **Q1** | **Q3** | **Min** | **Max** |
| --- | --- | --- | --- | --- | --- | --- | --- | --- | --- |
| **≥ 14 years** | **Low GES** | 516 | 52.8 | 50 | 15.4 | 42 | 62 | 7 | 115 |
|  | **Middle GES** | 493 | 56.5 | 54 | 17.3 | 45 | 66 | 26 | 171 |
|  | **High GES** | 476 | 60.1 | 58 | 17.4 | 47 | 71 | 25 | 135 |
| **11-13 years** | **Low GES** | 819 | 55.8 | 53 | 16.6 | 44 | 65 | 8 | 141 |
|  | **Middle GES** | 822 | 59 | 57 | 17.2 | 47 | 69 | 24 | 155 |
|  | **High GES** | 861 | 61.8 | 60 | 18.1 | 49 | 73 | 27 | 154 |
| **≤ 10 years** | **Low GES** | 175 | 57.5 | 55 | 17.8 | 45 | 68 | 28 | 127.6 |
|  | **Middle GES** | 176 | 60.3 | 58.5 | 16.6 | 47 | 72.5 | 18 | 113 |
|  | **High GES** | 164 | 61.6 | 61.5 | 16.5 | 49 | 71 | 28 | 117 |

**Figure S3** Description of HDL-C (mg/dl) in different combinations of GES_HDL-C_ tertiles based on [14] and a) education groups and b) income tertiles.


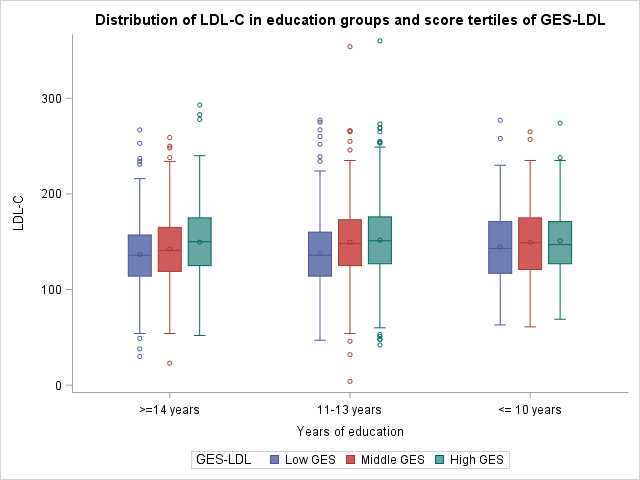

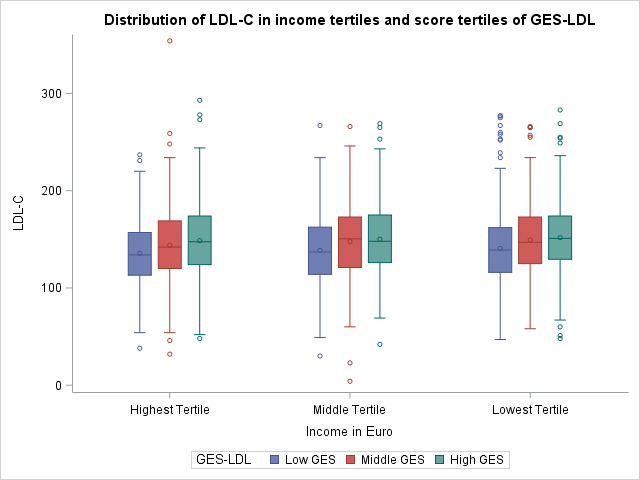


b

a

| **Education** | **GES_LDL-C_** | **N** | **Mean** | **Median** | **SD** | **Q1** | **Q3** | **Min** | **Max** |
| --- | --- | --- | --- | --- | --- | --- | --- | --- | --- |
| **≥ 14 years** | **Low GES** | 472 | 136.9 | 136 | 34.4 | 114 | 157 | 30 | 267 |
|  | **Middle GES** | 493 | 142.3 | 141 | 35.9 | 119 | 165 | 23 | 259 |
|  | **High GES** | 518 | 149.6 | 150 | 36.1 | 125 | 175 | 52 | 293 |
| **11-13 years** | **Low GES** | 843 | 137.5 | 136 | 33.5 | 114 | 160 | 47 | 277 |
|  | **Middle GES** | 816 | 149.5 | 148 | 36.8 | 125 | 173 | 4 | 354 |
|  | **High GES** | 834 | 151.7 | 151 | 37 | 127 | 176 | 42 | 360 |
| **≤ 10 years** | **Low GES** | 169 | 144.4 | 143 | 37.2 | 117 | 171 | 63 | 277 |
|  | **Middle GES** | 172 | 149.4 | 149 | 37.3 | 121 | 175 | 61 | 265 |
|  | **High GES** | 172 | 150.9 | 147 | 34.6 | 127 | 171 | 69 | 274 |

| **Income** | **GES_LDL-C_** | **N** | **Mean** | **Median** | **SD** | **Q1** | **Q3** | **Min** | **Max** |
| --- | --- | --- | --- | --- | --- | --- | --- | --- | --- |
| **Highest Tertile** | **Low GES** | 493 | 135.7 | 134 | 32.2 | 113 | 157 | 38 | 237 |
|  | **Middle GES** | 499 | 143.9 | 142 | 378 | 120 | 169 | 32 | 354 |
|  | **High GES** | 450 | 148.6 | 147.5 | 38.1 | 124 | 174 | 48 | 293 |
| **Middle Tertile** | **Low GES** | 396 | 138.6 | 137 | 33.7 | 114 | 162.5 | 30 | 267 |
|  | **Middle GES** | 386 | 147.6 | 150.5 | 36.8 | 121 | 173 | 4 | 266 |
|  | **High GES** | 454 | 150.2 | 148 | 35.3 | 126 | 175 | 42 | 269 |
| **Lowest Tertile** | **Low GES** | 509 | 140.5 | 139 | 36.8 | 116 | 162 | 47 | 277 |
|  | **Middle GES** | 501 | 149.2 | 147 | 35.9 | 125 | 173 | 58 | 266 |
|  | **High GES** | 532 | 151.7 | 151 | 34.6 | 129.5 | 174 | 48 | 283 |

**Figure S4** Description of LDL-C (mg/dl) in different combinations of GES_LDL-C_ tertiles based on [14] and a) education groups and b) income tertiles.

| **Income** | **GES_TC_** | **N** | **Mean** | **Median** | **SD** | **Q1** | **Q3** | **Min** | **Max** |
| --- | --- | --- | --- | --- | --- | --- | --- | --- | --- |
| **Highest Tertile** | **Low GES** | 514 | 218.3 | 217 | 35.6 | 195 | 239 | 101 | 425 |
|  | **Middle GES** | 469 | 228.4 | 226 | 39.4 | 202 | 251 | 134 | 440 |
|  | **High GES** | 463 | 234.8 | 234 | 39.8 | 206 | 261 | 129 | 358 |
| **Middle Tertile** | **Low GES** | 415 | 223 | 221 | 35.9 | 196 | 246 | 136 | 376 |
|  | **Middle GES** | 394 | 228.3 | 228 | 36.7 | 204 | 252 | 98 | 337 |
|  | **High GES** | 430 | 236.2 | 236 | 40.3 | 209 | 261 | 99 | 365 |
| **Lowest Tertile** | **Low GES** | 482 | 221.7 | 221 | 36.6 | 198 | 244 | 115 | 326 |
|  | **Middle GES** | 542 | 231.4 | 231 | 40.7 | 204 | 257 | 106 | 389 |
|  | **High GES** | 524 | 238.2 | 236 | 39.2 | 211.5 | 260.5 | 134 | 390 |


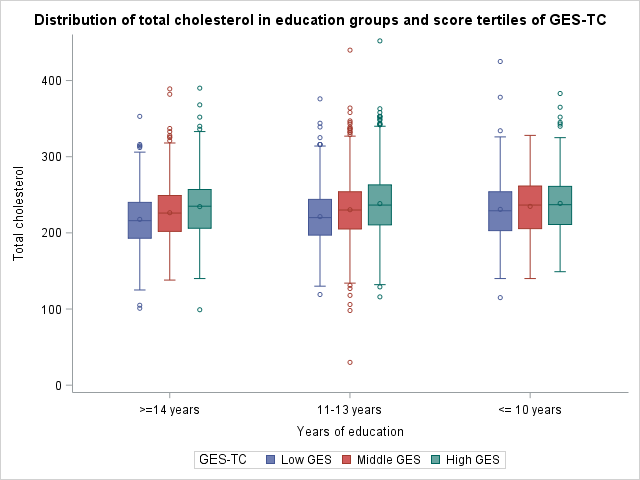

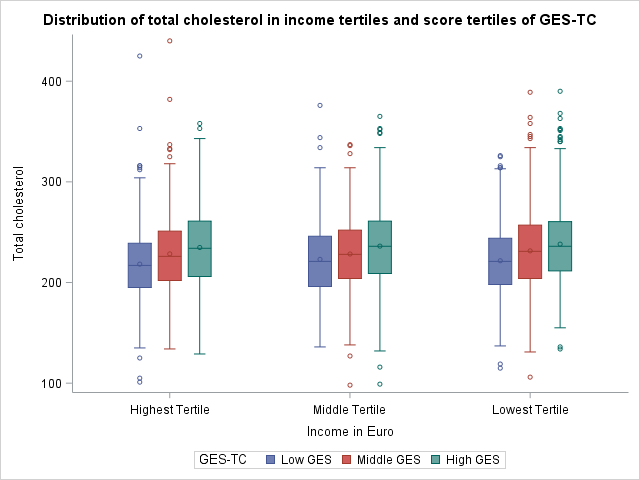


b

a

| **Education** | **GES_TC_** | **N** | **Mean** | **Median** | **SD** | **Q1** | **Q3** | **Min** | **Max** |
| --- | --- | --- | --- | --- | --- | --- | --- | --- | --- |
| **≥ 14 years** | **Low GES** | 508 | 217.6 | 216 | 34.9 | 193 | 240 | 101 | 353 |
|  | **Middle GES** | 473 | 226.4 | 226 | 37.7 | 202 | 249 | 138 | 389 |
|  | **High GES** | 505 | 234.4 | 235 | 40.1 | 206 | 257 | 99 | 390 |
| **11-13 years** | **Low GES** | 832 | 221.3 | 220 | 35.6 | 197 | 244 | 119 | 376 |
|  | **Middle GES** | 850 | 230.3 | 230 | 40.6 | 205 | 254 | 30 | 440 |
|  | **High GES** | 820 | 238.4 | 236.5 | 39.9 | 210.5 | 263 | 116 | 452 |
| **≤ 10 years** | **Low GES** | 162 | 230.9 | 229 | 42.6 | 203 | 254 | 115 | 425 |
|  | **Middle GES** | 172 | 234.8 | 236.5 | 37.3 | 205.5 | 261.5 | 140 | 328 |
|  | **High GES** | 181 | 238.7 | 237 | 41.4 | 211 | 261 | 149 | 383 |

**Figure S5** Description of TC (mg/dl) in different combinations of GES_TC_ tertiles based on [14] and a) education groups and b) income tertiles.

**ß GES_LDL-C_**

**Figure S6** Sex-, age-adjusted and effects per GES_LDL-C_ standard deviation and corresponding 95% confidence interval (95% CI) of the genetic effect on low-density lipoprotein cholesterol (LDL-C), stratified by diabetes mellitus in linear regression models using the GES_LDL-C_ based on [14].
